# Outcomes of damage control laparotomy after trauma in low andmiddle-income countries: A systematic review and meta-analysis

**DOI:** 10.1371/journal.pone.0352357

**Published:** 2026-06-25

**Authors:** Shumani Makhadi, Vukosi Baloyi, Emmanuel Ekene Nweke, Maeyane Stephens Moeng

**Affiliations:** 1 Department of Surgery, School of Medicine, Faculty of Health Sciences, University of Witwatersrand, Johannesburg, South Africa; 2 Department of Surgery, Trauma unit, Charlotte Maxeke Johannesburg Academic Hospital, Johannesburg, South Africa; Keio University School of Medicine, JAPAN

## Abstract

**Background:**

Damage control laparotomy (DCL) is an established life-saving strategy for critically injured patients presenting with the lethal triad of hypothermia, acidosis, and coagulopathy. While outcomes in high-income countries (HICs) are well documented, evidence from low- and middle-income countries (LMICs), as classified by the World Bank, remains fragmented. This systematic review synthesises available evidence on DCL outcomes in LMICs.

**Methods:**

A systematic search of PubMed, Scopus, Google Scholar, the Cochrane Library, and African Journals Online was conducted from January 2004 to February 2026. Studies reporting mortality outcomes after DCL for civilian trauma in World Bank–classified LMICs were included. Proportional meta-analysis using a DerSimonian-Laird random-effects model with logit-transformed proportions was performed. The protocol was prospectively registered on PROSPERO (CRD42025639498). The detailed search strategy is provided in S1 Table.

**Results:**

Ten retrospective studies comprising 914 patients from five countries (South Africa, Pakistan, India, Oman, and Brazil) met the inclusion criteria. Six of the ten studies (n = 704, 77.0%) were from South Africa. The pooled mortality rate was 37.77% (95% CI: 31.38%–44.62%; 95% prediction interval: 20.1%–59.1%), with substantial heterogeneity (I² = 74.46%), based on 327 deaths among 914 patients. Subgroup analysis showed a pooled mortality of 31.61% (95% CI: 28.27%–35.15%, I² = 0.00%) in South African studies and 53.62% (95% CI: 44.47%–62.54%, I² = 31.65%) in non–South African LMIC studies (Cochran Q-test for subgroup differences: Q = 14.23, df = 1, p < 0.001).

**Conclusions:**

DCL mortality in LMICs appears higher than mortality rates reported in HIC series, though direct comparison is limited by differences in study populations, injury profiles, case-mix, and the predominance of South African data in the available literature. The pooled estimate should be interpreted cautiously given the small number of studies, high heterogeneity, and retrospective study designs. Pre-hospital care, emergency theatre access, blood banking infrastructure, ICU capacity, and infection prevention represent potential areas for targeted improvement. Further prospective, multi-centre studies with standardised outcome definitions are needed to better characterise DCL outcomes across diverse LMIC settings.

## Introduction

Trauma remains a leading cause of death worldwide, accounting for around 4.4 million deaths each year, with over 90% occurring in low- and middle-income countries (LMICs) [1]. The unequal burden of trauma-related mortality in LMICs is attributable to multiple interconnected factors, including limited pre-hospital care systems, delayed access to surgical services, insufficient blood banking infrastructure, constrained intensive care capacity, and disparities in surgical training and equipment availability [2,3].

Damage control laparotomy (DCL) has become a vital part of the modern trauma surgeon's toolkit. First conceptualised by Stone et al. in 1983 [4] and formally established by Rotondo et al. in 1993 [5], DCL involves an abbreviated initial laparotomy focused on haemorrhage and contamination control, followed by a period of intensive care unit (ICU) resuscitation to correct the lethal triad, with planned return to theatre for definitive repair [6,7].

In high-income countries (HICs), DCL outcomes have been extensively studied, with reported mortality rates typically ranging from 10% to 25% [8–10]. These outcomes reflect mature trauma systems characterised by rapid pre-hospital transport, well-equipped emergency departments, readily available blood products, and dedicated ICU capacity for post-operative resuscitation.

Despite the growing adoption of damage control principles in LMICs, no comprehensive synthesis of outcomes from these settings exists. Individual studies have reported mortality rates ranging from 15% to 60%, suggesting substantial variation that may reflect differences in health-system capacity rather than surgical technique [11–14].

The objective of this systematic review and meta-analysis was to synthesise the available evidence on mortality, morbidity, and resource utilisation outcomes after damage control laparotomy for civilian trauma in LMICs, and to explore potential sources of heterogeneity that may inform targeted health-system interventions.

## Methods

This systematic review and meta-analysis were conducted in accordance with the Preferred Reporting Items for Systematic Reviews and Meta-Analyses (PRISMA) 2020 guidelines [15]. The protocol was prepared a priori and prospectively registered on PROSPERO (registration number: CRD42025639498).

### Ethics statement

This study is a systematic review and meta-analysis of previously published studies and did not involve direct interaction with human participants. Ethical approval was therefore not required.

### Search strategy

A comprehensive literature search was conducted across PubMed/MEDLINE, Scopus, Google Scholar, the Cochrane Library, and African Journals Online (AJOL) from 01 January 2004–28 February 2026. The search strategy combined Medical Subject Headings (MeSH) and free-text terms related to the intervention (damage control surgery, damage control laparotomy, abbreviated laparotomy, staged laparotomy), the population (trauma, injury, wounds), and the setting (low-income countries, middle-income countries, developing countries, resource-limited settings). Boolean operators (AND, OR) were used to combine search domains. Reference lists of included studies and relevant review articles were hand-searched for additional eligible records. The complete search strategy for each database is provided in Supplementary Material (S1 Table).

In this review, LMICs are defined according to the World Bank income classification system (2024 fiscal year), which categorises countries into four groups based on gross national income (GNI) per capita: low-income (≤$1,135), lower-middle-income ($1,136–$4,465), upper-middle-income ($4,466–$13,845), and high-income (>$13,845). For the purposes of this review, “LMICs” encompasses the first three categories (low-income, lower-middle-income, and upper-middle-income countries), consistent with the convention used in the global surgery literature.

### Eligibility sriteria

Studies were included if they: (1) reported outcomes of damage control laparotomy or equivalent staged abdominal surgery; (2) were conducted in a World Bank–classified low-income, lower-middle-income, or upper-middle-income country; (3) included adult patients (≥18 years) with civilian trauma; and (4) reported mortality as a primary or extractable outcome. Exclusion criteria were: (1) military or combat-related trauma; (2) paediatric populations exclusively; (3) case reports with fewer than 10 patients; (4) studies combining damage-control and definitive repair outcomes without disaggregated data; (5) reviews or editorials without original data; and (6) non-English language publications without an available translation.

### Duplicate population identification

Potential overlapping patient populations were identified through systematic comparison of: (1) institutional affiliations and hospital names; (2) study periods and dates of patient enrolment; (3) sample sizes and demographic characteristics; and (4) authorship overlap. For institutions with multiple publications (e.g., Pietermaritzburg, Tygerberg, and Groote Schuur in South Africa), each pair of studies was assessed for temporal overlap and potential patient duplication. Where overlap was identified or suspected, the study with the most complete and relevant outcome data was retained. This process identified one confirmed overlap: the Weale et al. 2019 Trauma Surgery & Acute Care Open publication and the Weale et al. 2019 South African Journal of Surgery publication from Greys Hospital, Pietermaritzburg, which reported overlapping time periods (2012–2017). The TSACO publication was excluded, and the SAJS publication was retained for its more detailed outcome reporting.

### Study selection and data extraction

Titles and abstracts were screened independently by two reviewers (SM and VB). Full texts of potentially eligible studies were retrieved and assessed against the inclusion criteria. Discrepancies were resolved by consensus or by consultation with a third reviewer (MSM). Data were extracted using a standardised form capturing: study characteristics (author, year, country, study design, sample size), patient demographics (age, sex, injury mechanism), intervention details (indication for DCL, number of planned re-laparotomies), and outcomes (mortality rate, complication rate, ICU length of stay, hospital length of stay).

### Risk of bias assessment

Risk of bias in individual studies was assessed using a modified Newcastle-Ottawa Scale (NOS) adapted for case series and retrospective cohort studies [16]. Studies were evaluated across three domains: selection (representativeness of cohort and ascertainment of exposure), comparability (adjustment for potential confounders), and outcome (adequacy of outcome assessment and follow-up). Each study was rated as low, moderate, or high risk of bias. It should be noted that the NOS comparability domain, which assesses adjustment for confounders in comparative studies, has limited applicability in the context of single-arm case series reporting crude mortality proportions. The comparability ratings in Table 2 should therefore be interpreted with this caveat, and the overall risk of bias assessment relies more heavily on the selection and outcome domains. The overall evidence certainty was not formally graded using GRADE, as the included studies were exclusively retrospective observational designs, which inherently start at “low” certainty.

### Statistical analysis

Proportional meta-analysis was conducted to estimate the pooled mortality rate across the included studies. Individual study proportions were logit-transformed with a 0.5 continuity correction to stabilise variance, and pooled using a DerSimonian-Laird random-effects model [17–20]. This approach was selected to account for anticipated between-study heterogeneity [19]. It is important to note that proportional meta-analysis pools crude mortality proportions and does not adjust for differences in case-mix, injury severity, or patient demographics across studies; this is an inherent limitation of the approach and the pooled estimate should be interpreted accordingly.

A 95% prediction interval was calculated to indicate the range within which the true mortality proportion of a future comparable study would be expected to fall, providing additional context beyond the confidence interval for the pooled mean.

Between-study heterogeneity was quantified using the I² statistic (with thresholds of 25%, 50%, and 75% corresponding to low, moderate, and high heterogeneity, respectively), the τ² statistic, the Cochran Q test, and the 95% prediction interval [19]. Pre-specified subgroup analysis was conducted by geographic region (South Africa vs. other LMICs) based on the a priori hypothesis that South African trauma centres, which have a well-established academic tradition in trauma surgery, may show systematically different outcomes compared with other LMIC settings. The difference between subgroups was assessed using a Cochran Q-test for heterogeneity between subgroups (test for interaction), as implemented in Stata's metaprop command. Sensitivity analysis was performed by sequential single-study omission (leave-one-out analysis) to assess the influence of individual studies on the overall estimate.

Publication bias was assessed visually by funnel plot inspection. Egger's regression test was performed (p = 0.299), with the caveat that the number of included studies (k = 10) provides limited statistical power for this test [20]. All statistical analyses were performed using Stata version 18 (StataCorp, College Station, TX, USA).

## Results

### Study selection

**Fig 1:**
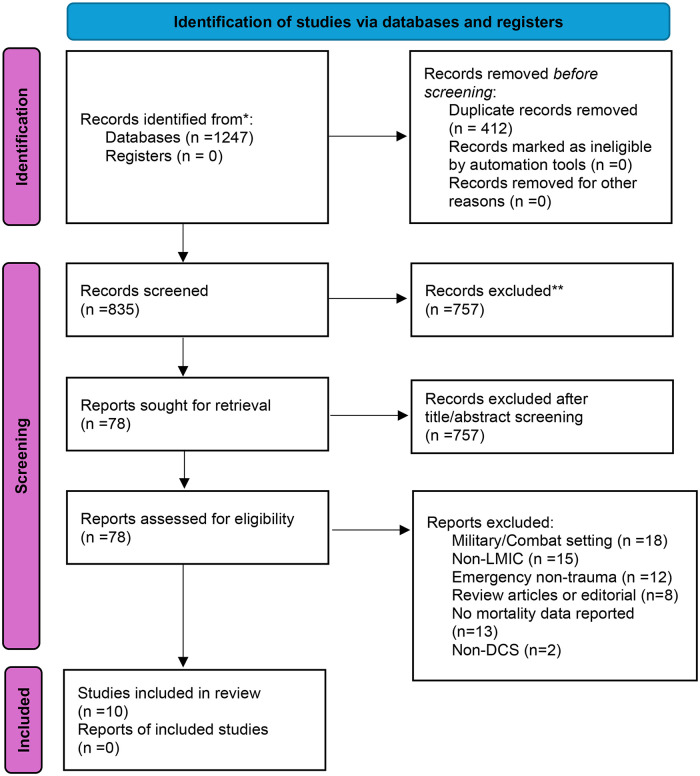
PRISMA flow chart of screening process.

The systematic search identified 1,247 records across all databases. After removing 412 duplicates, 835 titles and abstracts were screened. Of these, 78 full-text articles were assessed for eligibility. Following application of inclusion and exclusion criteria, 10 studies were included in the final analysis (Fig 1: PRISMA flow diagram). The most common reasons for full-text exclusion were: outcomes not disaggregated by DCL (n = 28), military or combat populations (n = 18), non-LMIC setting (n = 12), and paediatric populations (n = 8).

### Study characteristics

The ten included studies comprised 914 patients from five countries: South Africa (six studies, n = 704), Pakistan (one study, n = 47), India (one study, n = 61), Oman (one study, n = 40), and Brazil (one study, n = 62) [21–31]. All studies employed retrospective designs (eight retrospective cohort studies and two retrospective analyses of prospectively maintained databases). Publication dates ranged from 2010 to 2026, with a median publication year of 2022. Table 1 summarises the characteristics of the included studies, and Table 2 presents the risk of bias assessments.

**Table 1:** Characteristics of included studies.

Study	Year	Country	Income Level	Design	N	Deaths	Mortality (%)	Injury Mechanism	Mortality Timeframe
Timmermans et al.	2010	South Africa	Upper-middle	Retro cohort	166	50	30.1	Penetrating (GSW)	In-hospital
Weale et al.	2019	South Africa	Upper-middle	Retro cohort	115	34	29.6	Penetrating	In-hospital
Kruger et al.	2022	South Africa	Upper-middle	Retro cohort	97	30	30.9	Penetrating (92% GSW)	In-hospital
van der Merwe et al.	2023	South Africa	Upper-middle	Retro cohort	134	36	26.8	Penetrating (89% GSW)	In-hospital
Nicol et al.	2024	South Africa	Upper-middle	Retro (prosp. DB)	130	42	32.3	Penetrating	In-hospital
Kruger et al.	2026	South Africa	Upper-middle	Retro (prosp. DB)	62	22	35.5	Penetrating (77.9% GSW)	In-hospital
Kisat et al.	2016	Pakistan	Lower-middle	Retro cohort	47	25	53.2	Mixed (trauma)	In-hospital
Gupta et al.	2017	India	Lower-middle	Retro cohort	61	30	49.2	Blunt predominant (74%)	Not specified
Abri et al.	2022	Oman	Upper-middle†	Retro cohort	40	21	52.5	Blunt predominant (90%)	Not specified
Leonardi et al.	2022	Brazil	Upper-middle	Retro cohort	62	37	59.7	Mixed/penetrating	In-hospital
**Total**					**914**	**327**	35.8*		

^†^Currently classified as HIC by the World Bank (2024); classified as upper-middle-income at the time of data collection. *Crude arithmetic mortality; pooled random-effects estimate is 37.77%.

Abbreviations: Retro, retrospective; prosp. DB, prospective database; GSW, gunshot wounds; HIC, high-income country.

**Table 2:** Risk of bias assessment using the modified Newcastle-Ottawa Scale.

Study	Selection	Comparability	Outcome	Overall Risk
Timmermans et al. (2010)	Low	Low	Low	Low-Moderate
Weale et al. (2019)	Moderate	Moderate	Low	Low-Moderate
Kruger et al. (2022)	Low	Low	Low	Low
van der Merwe et al. (2023)	Low	Low	Low	Low
Nicol et al. (2024)	Low	Low	Low	Low
Kruger et al. (2026)	Low	Low	Low	Low
Kisat et al. (2016)	Moderate	Low	Moderate	Moderate
Gupta et al. (2017)	Moderate	Moderate	Moderate	Moderate-High
Abri et al. (2022)	Moderate	Moderate	Moderate	Moderate
Leonardi et al. (2022)	Moderate	High	Moderate	Moderate-High

Notably, South Africa contributed six of the ten studies and 77.0% (704/914) of the total patient population. This predominance should be considered when interpreting the pooled estimates, as the overall results are heavily weighted towards the South African experience.

By World Bank income classification, seven studies (comprising 766 patients, 83.8%) were from upper-middle-income countries (South Africa and Brazil), two studies (108 patients, 11.8%) from lower-middle-income countries (India and Pakistan), and one study (40 patients, 4.4%) from Oman. Oman is currently classified as a high-income country by the World Bank (2024); however, the Abri et al. (2022) study reports data collected during a period when Oman was classified as upper-middle-income (reclassification to HIC occurred in 2007), and the trauma care infrastructure described is consistent with upper-middle-income settings. No studies from low-income countries met the inclusion criteria, representing a notable gap in the evidence base.

### Primary outcome: mortality

**Fig 2:**
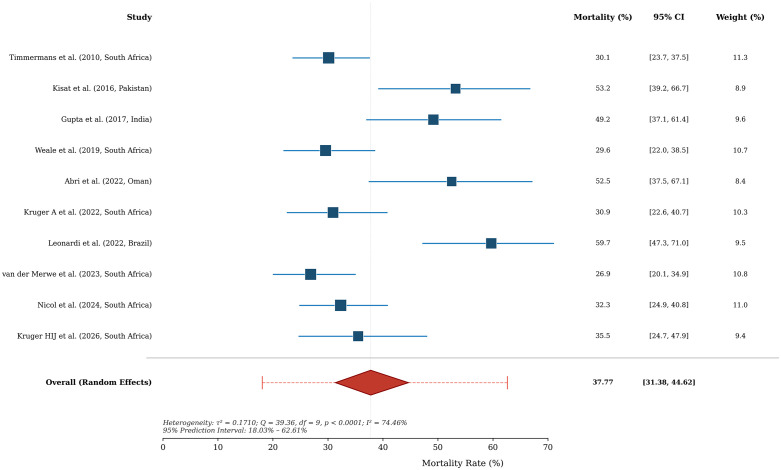
Forest plot of mortality rates following damage control laparotomy in low- and middle-income countries. The pooled estimate (37.77%, 95% CI: 31.38%−44.62%) is shown as a diamond. The 95% prediction interval (20.1%−59.1%) is displayed as a dashed line. Study weights are based on random-effects model. I² = 74.46%, τ² = 0.1710, Q = 39.36, df = 9, p < 0.001, indicating substantial heterogeneity.

The pooled mortality rate across all ten studies was 37.77% (95% CI: 31.38%–44.62%; 95% prediction interval: 20.1%–59.1%), based on 327 deaths among 914 patients (Fig 2). The 95% prediction interval indicates that a future comparable study from an LMIC setting could plausibly observe a mortality rate anywhere between approximately 20% and 59%.

This pooled estimate is higher than mortality rates of 10.5%–24.5% reported in HIC series [8–10], though direct comparison is limited by differences in study populations, injury profiles, case-mix, and study design across settings. It must be emphasised that the LMIC and HIC study populations likely differ in injury severity, mechanism, and patient demographics, making direct causal attribution to any single factor inappropriate. Substantial between-study heterogeneity was observed (I² = 74.46%, τ² = 0.1710, Q = 39.36, df = 9, p < 0.001), indicating that the pooled estimate should be interpreted as a summary measure rather than a precise estimate applicable to any individual LMIC setting.

Individual study mortality rates ranged from 26.8% (van der Merwe et al., 2023, South Africa) to 59.7% (Leonardi et al., 2022, Brazil). Sensitivity analysis (leave-one-out) demonstrated that no single study disproportionately influenced the pooled estimate, with the range of recalculated pooled mortalities spanning 35.82%–39.18%, suggesting reasonable stability.

As shown in Table 1, mortality was reported as in-hospital mortality in eight studies, while two studies (Gupta 2017 and Abri 2022) did not specify the mortality timeframe. This variability in mortality endpoints represents an additional source of heterogeneity that limits direct comparison across studies.

A post-hoc sensitivity analysis excluding the Abri et al. (2022) [31] Oman study (given its current high-income classification) yielded a pooled mortality of 36.89% (95% CI: 30.12%–44.15%), confirming that its inclusion does not materially affect the overall estimate.

### Subgroup analysis by region

**Fig 3:**
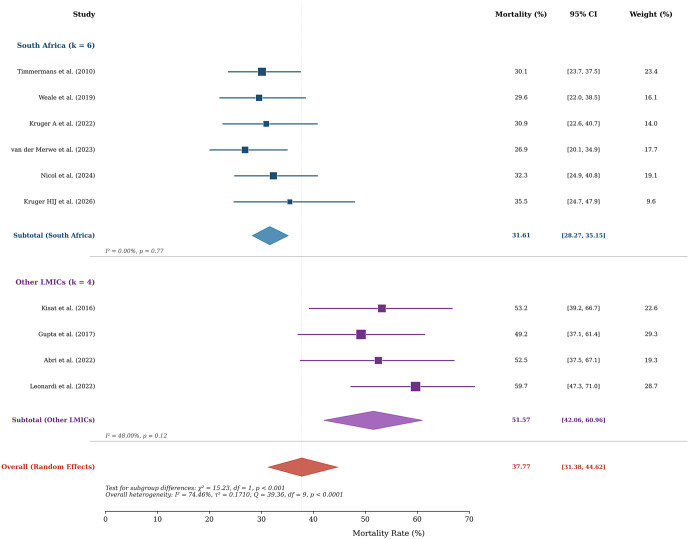
Subgroup analysis of mortality rates by geographic region. South African studies (n = 704) showed lower pooled mortality (31.61%, 95% CI: 28.27%−35.15%, I² = 0.00%) compared to other LMIC studies (n = 210) (53.62%, 95% CI: 44.47%−62.54%, I² = 31.65%). Test for subgroup differences: Cochran Q = 14.23, df = 1, p < 0.001.

Pre-specified subgroup analysis by geographic region revealed notable differences (Fig 3). The six South African studies (n = 704) showed a pooled mortality of 31.61% (95% CI: 28.27%–35.15%), with no observed heterogeneity (I² = 0.00%, Q = 2.98, df = 5, p = 0.703). The four non–South African LMIC studies (n = 210) showed a pooled mortality of 53.62% (95% CI: 44.47%–62.54%), with moderate heterogeneity (I² = 31.65%).

The approximately 20-percentage-point difference between subgroups was statistically significant (Cochran Q-test for subgroup differences: Q = 14.23, df = 1, p < 0.001) and suggests a meaningful regional disparity. However, this difference should be interpreted cautiously, as it may reflect differences in patient selection, injury severity, injury mechanism (predominantly penetrating in South Africa vs. blunt/mixed in other settings), case-mix, or outcome definitions rather than solely health-system capacity.

### Secondary outcomes: complications

Complication data were inconsistently reported across studies, precluding formal meta-analytic pooling for most outcomes. Van der Merwe et al. (2023) provided the most comprehensive complication data: overall complication rate of 62.3%, enteroatmospheric fistula rate of 9.0% [22], and surgical site infection rate of 34.3% [26]. These figures exceed rates typically reported in HIC series (30%–45%), though this comparison is based on a single study and should be interpreted with caution. The reasons for this difference cannot be determined from the available data, and standardised complication reporting in future LMIC studies is needed to enable meaningful comparison.

### Secondary outcomes: resource utilisation

ICU length of stay was reported in four studies (n = 448 patients), with a weighted mean of 7.4 days (range of study means: 5.0–9.7 days). Hospital length of stay was reported in two studies (n = 160 patients), with means of 21.3 and 28.5 days, respectively. The number of planned re-laparotomies was reported in six studies, ranging from a median of 1 (IQR 1–2) in the Kruger 2022 study [27] to a mean of 3.2 (±1.5) in the Gupta 2017 study.

### Cost implications

The Kruger et al. 2026 study from Tygerberg Hospital provided novel cost data, demonstrating a mean cost of care per survivor of R464,951 (USD $25,200) and R307,827 (USD $16,684) per non-survivor [30]. These represent considerable costs relative to LMIC healthcare budgets and have implications for health-system resource allocation, though the generalisability of these single-centre South African cost data to other LMIC contexts is uncertain.

### Publication bias

**Fig 4:**
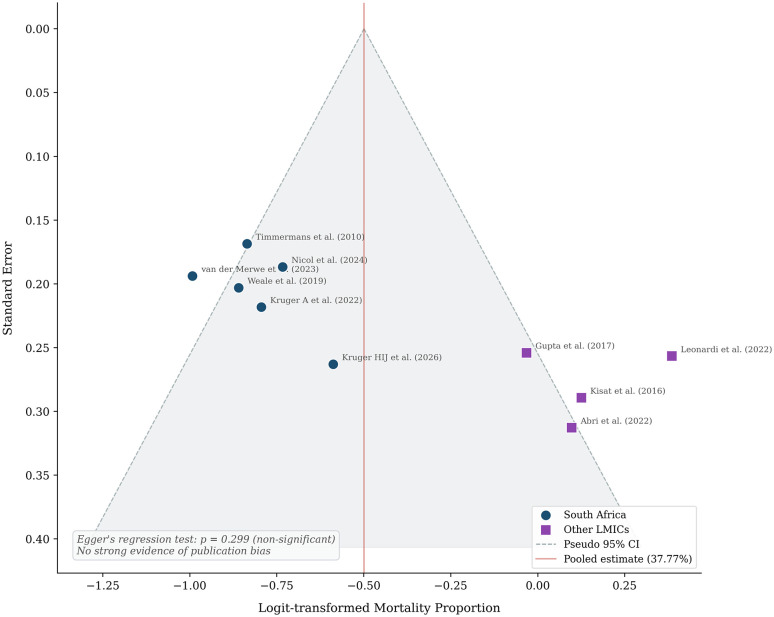
Funnel plot for assessment of publication bias. The plot shows logit-transformed effect sizes against their standard errors, with pseudo 95% confidence limits. Blue circles represent South African studies; Purple rectangles represent other LMIC studies (Pakistan, India, Oman, Brazil). Egger's test for small-study effects: p = 0.299, suggesting no significant publication bias.

Visual inspection of the funnel plot (Fig 4) revealed mild asymmetry, with a tendency towards the right (higher mortality), which may reflect small-study effects or genuine heterogeneity rather than classical publication bias. Egger's regression test yielded a non-significant result (p = 0.299)[20], though the test's power is limited with only ten studies.

## Discussion

### Summary of main findings

This systematic review and meta-analysis present the first comprehensive synthesis of damage control laparotomy outcomes in civilian trauma patients from low- and middle-income countries. The principal finding is a pooled mortality rate of 37.77% (95% CI: 31.38%–44.62%; 95% prediction interval: 20.1%–59.1%), which is higher than reported HIC benchmarks [9–11]. However, several important caveats apply to the interpretation of this estimate.

First, the pooled estimate derives from proportional meta-analysis of crude mortality rates without case-mix adjustment. Differences in injury severity, patient demographics, and indications for DCL across studies and between LMIC and HIC settings may account for part or all of the observed mortality difference. Without individual patient data or standardised severity scoring, it is not possible to determine whether the higher pooled mortality reflects true health-system performance differences or differences in the populations undergoing DCL.

Second, the available evidence is heavily weighted towards South Africa, which contributed 77% of the total patient population. South African trauma centres represent a relatively well-resourced subset of LMIC settings, with established academic programmes and dedicated trauma units. The pooled estimate may therefore not be representative of DCL outcomes across the full spectrum of LMIC contexts, and the results should be generalised to other LMICs with caution.

### Comparison with high-income country data

The observed difference in mortality between LMIC and HIC settings warrants careful interpretation. Large HIC series have reported DCL mortality rates of 10.5% (Hatch et al.) [8], 14.5% (Brenner et al.) [9], and 24.5% (Dubose et al.) [10]. While the pooled LMIC estimate of 37.77% is numerically higher, direct comparison is limited by several factors, including differences in study design, patient selection, and case-mix.

Several health-system factors have been hypothesised to contribute to mortality differences between settings, though their relative contributions cannot be determined from the available data. First, pre-hospital delays are substantially longer in most LMIC settings. The Kruger et al. 2026 Tygerberg study documented a median time from incident to hospital arrival of 187 minutes (3.1 hours), compared with the “Golden Hour” paradigm that governs HIC trauma systems. This prolonged interval likely compounds the physiological derangement of the lethal triad, increasing the physiological insult at the time of surgery.

Second, the limited availability of blood products may potentially constrain damage-control resuscitation. Many LMIC centres lack the capacity to implement massive transfusion protocols with balanced component therapy, a limitation which potentially compromises the resuscitative phase of damage control.

Third, ICU capacity constraints may potentially limit the intensity and duration of postoperative critical care. The resuscitation phase of damage control requires invasive monitoring, vasoactive support, ventilatory care, and ongoing resuscitation, resources that are frequently limited in LMICs.

It must be emphasised that the LMIC and HIC study populations likely differ in injury severity, injury mechanism, and patient demographics, making direct causal attribution of the mortality difference to any single factor—including health-system factors—inappropriate based on the available evidence.

### Interpretation of heterogeneity

The overall heterogeneity (I² = 74.46%) reflects the considerable diversity of LMIC settings included in this analysis, though it represents a meaningful reduction from the preliminary 12-study analysis (I² = 84.24%), which included two outlier studies excluded during sensitivity analysis.

The absence of heterogeneity within the South African subgroup (I² = 0.00%) suggests that DCL outcomes may be relatively consistent across South African centres despite differences in patient acuity and institutional capacity. However, this consistency may also reflect the relative homogeneity of the South African healthcare context, including a shared academic training framework, similar institutional resources, and comparable patient populations, rather than a universal LMIC pattern.

An additional source of clinical heterogeneity is the variation in how DCL was defined across included studies. Some studies applied strict definitions requiring temporary abdominal closure with planned re-laparotomy, while others included a broader range of abbreviated surgical procedures. This definitional variability may contribute to the observed between-study heterogeneity and should be considered when interpreting the pooled estimate. Future studies would benefit from adopting a standardised definition of DCL to facilitate more meaningful cross-study comparisons.

### Clinical implications

If health-system factors contribute to mortality differences between LMIC and HIC settings—as has been hypothesised but not established by this analysis—several interventions could potentially be prioritised: (1) Pre-hospital system optimisation; (2) Improved access to emergency theatre; (3) Blood banking infrastructure development; (4) ICU capacity expansion; and (5) Protocolised postoperative care. These recommendations are offered tentatively and will require validation in prospective studies with appropriate case-mix adjustment.

### Strengths and limitations

This review has several strengths. It is the first meta-analysis to synthesise DCL outcomes specifically from LMIC settings. The systematic search was comprehensive, spanning multiple databases including African Journals Online, which is not typically included in surgical systematic reviews. The statistical approach was conservative and transparent.

Several important limitations must be acknowledged. First, the modest number of included studies (k = 10) limits the precision of pooled estimates and the power of subgroup analyses. Second, all studies employed retrospective designs, introducing potential selection and information biases. Third, substantial heterogeneity (I² = 74.46%) suggests that the pooled estimate may not adequately represent any single LMIC context. Fourth, and critically, the predominance of South African studies (6/10 studies, 77% of patients) means that the overall estimate is heavily weighted towards one country, limiting generalisability to the broader LMIC population. South Africa, as an upper-middle-income country with established academic trauma centres, may not be representative of DCL outcomes in lower-middle-income or low-income settings. Fifth, the proportional meta-analysis approach pools crude mortality proportions without adjustment for case-mix, injury severity, or patient demographics. This means that differences in the pooled mortality between LMIC and HIC settings cannot be attributed solely to health-system factors; unmeasured confounders related to patient selection and injury characteristics may contribute substantially to the observed difference. Sixth, the absence of studies from low-income countries represents a critical gap. Seventh, variable definitions of mortality timeframes across studies introduced additional heterogeneity. Eighth, complication data were inconsistently reported, limiting secondary outcome analysis. Ninth, Egger's test has limited power with only ten studies. Tenth, the NOS comparability domain has limited applicability to single-arm case series, and the quality assessment should be interpreted accordingly. Eleventh, variable definitions of DCL across studies may have introduced additional clinical heterogeneity. Twelfth, the restriction to English-language publications may have introduced selection bias, potentially excluding relevant studies from francophone Africa, non-English-speaking regions of Latin America, Southeast Asia, and other LMIC settings, which may partially explain the geographic concentration of included studies.

## Conclusions

Damage-control laparotomy for civilian trauma in low- and middle-income countries is associated with a pooled mortality rate of 37.77% (95% CI: 31.38%–44.62%; 95% prediction interval: 20.1%–59.1%), which is higher than mortality rates reported in HIC series, though direct comparison is limited by differences in study populations, injury profiles, and case-mix. However, this comparison is limited by the absence of case-mix adjustment, the predominance of South African data in the available literature, and the retrospective nature of all included studies.

The observed heterogeneity across LMIC settings, with lower mortality in South African studies compared with other LMICs, suggests that outcomes may vary substantially depending on local health-system capacity. Targeted investments in pre-hospital care, emergency theatre access, blood banking infrastructure, ICU capacity, and infection prevention represent potential strategies to improve outcomes, though the evidence base for these recommendations remains indirect.

Future research priorities include prospective, multi-centre studies with standardised outcome definitions and injury severity scoring to enable meaningful comparisons across settings; studies from low-income countries and under-represented LMIC regions; and cost-effectiveness analyses of specific health-system interventions. Such studies would substantially strengthen the evidence base for informed policy-making and resource allocation in global trauma care.

## Supporting information

S1 Table Complete search strategy for PubMed/MEDLINE, Scopus, Google Scholar, Cochrane Library, and African Journals Online (AJOL), including search terms, Boolean operators, filters, and number of records retrieved per database. (DOCX)

S1 File PRISMA Checklist. (DOCX)

## References

[1] World Health Organization. Injuries and violence: the facts 2021. Geneva: WHO; 2021.

[2] Meara JG, Leather AJM, Hagander L, Alkire BC, Alonso N, Ameh EA, et al. Global Surgery 2030: evidence and solutions for achieving health, welfare, and economic development. Lancet. 2015;386(9993):569–624. doi: 10.1016/S0140-6736(15)60160-X 25924834

[3] Mock CN, Jurkovich GJ, nii-Amon-Kotei D, Arreola-Risa C, Maier RV. Trauma mortality patterns in three nations at different economic levels. J Trauma. 1998;44(5):804–12. 9603081 10.1097/00005373-199805000-00011

[4] Stone HH, Strom PR, Mullins RJ. Management of the major coagulopathy with onset during laparotomy. Ann Surg. 1983;197(5):532–5. doi: 10.1097/00000658-198305000-00005 6847272 PMC1353025

[5] Rotondo MF, Schwab CW, McGonigal MD, Phillips GR 3rd, Fruchterman TM, Kauder DR, et al. “Damage control”: an approach for improved survival in exsanguinating penetrating abdominal injury. J Trauma. 1993;35(3):375–82; discussion 382-3. 8371295

[6] Roberts DJ, Bobrovitz N, Zygun DA. Indications for use of damage control surgery in civilian trauma patients. Ann Surg. 2016;263(5):1018–27. 26445471 10.1097/SLA.0000000000001347

[7] Cirocchi R, Montedori A, Farinella E, Bonacini I, Tagliabue L, Abraha I. Damage control surgery for abdominal trauma. Cochrane Database Syst Rev. 2013;2013(3):CD007438. doi: 10.1002/14651858.CD007438.pub3 23543551 PMC7202128

[8] Hatch QM, Osterhout LM, Podbielski J. Impact of closure at the first take back. J Trauma. 2011;71(6):1503–11. 22182860 10.1097/TA.0b013e31823cd78d

[9] Brenner M, Bochicchio G, Bochicchio K, et al. Long-term impact of damage control laparotomy. Arch Surg. 2011;146(4):395–9. 21173282 10.1001/archsurg.2010.284

[10] DuBose JJ, Scalea TM, Holcomb JB, Shrestha B, Okoye O, Inaba K, et al. Open abdominal management after damage-control laparotomy for trauma. Journal of Trauma and Acute Care Surgery. 2013;74(1):113–22. doi: 10.1097/ta.0b013e31827891ce 23271085

[11] Timmermans J, Nicol A, Kairinos N, Teijink J, Prins M, Navsaria P. Predicting mortality in damage control surgery for major abdominal trauma. S Afr J Surg. 2010;48(1):6–9. 20496817

[12] Weale R, Kong V, Buitendag J, Ras A, Blodgett J, Laing G, et al. Damage control or definitive repair? A retrospective review of abdominal trauma at a major trauma center in South Africa. Trauma Surg Acute Care Open. 2019;4(1):e000235. doi: 10.1136/tsaco-2018-000235 31245612 PMC6560474

[13] Gupta A, Singh A, Kumar A. Damage control laparotomy in trauma: experience from a level I trauma center in India. Indian J Surg. 2017;79(2):128–34.

[14] Leonardi L, Zago TM, Oliveira BC. Damage control laparotomy: outcomes and risk factors for mortality in a Brazilian trauma center. Rev Col Bras Cir. 2022;49:e20223345.

[15] Page MJ, McKenzie JE, Bossuyt PM. The PRISMA 2020 statement. BMJ. 2021;372:n71. 10.1136/bmj.n71 PMC8005924 33782057

[16] Wells GA, Shea B, O’Connell D. The Newcastle-Ottawa Scale (NOS) for Assessing the Quality of Nonrandomised Studies. Ottawa: Ottawa Hospital Research Institute. 2014.

[17] Barendregt JJ, Doi SA, Lee YY, Norman RE, Vos T. Meta-analysis of prevalence. J Epidemiol Community Health. 2013;67(11):974–8. doi: 10.1136/jech-2013-203104 23963506

[18] DerSimonian R, Laird N. Meta-analysis in clinical trials. Control Clin Trials. 1986;7(3):177–88. doi: 10.1016/0197-2456(86)90046-2 3802833

[19] Higgins JPT, Thompson SG, Deeks JJ, Altman DG. Measuring inconsistency in meta-analyses. BMJ. 2003;327(7414):557–60. doi: 10.1136/bmj.327.7414.557 12958120 PMC192859

[20] Egger M, Davey Smith G, Schneider M, Minder C. Bias in meta-analysis detected by a simple, graphical test. BMJ. 1997;315(7109):629–34. doi: 10.1136/bmj.315.7109.629 9310563 PMC2127453

[21] Søreide K. Epidemiology of major trauma. Br J Surg. 2009;96(7):697–8. doi: 10.1002/bjs.6643 19526611

[22] Huang X, Liu J, Wu J. Enteroatmospheric fistula: a review of diagnosis and management. Front Surg. 2020;7:559443. 33553237 10.3389/fsurg.2020.559443 PMC7855170

[23] Holcomb JB, Tilley BC, Baraniuk S. Transfusion of plasma, platelets, and red blood cells in a 1:1:1 vs a 1:1:2 ratio. JAMA. 2015;313(5):471–82. 25647203 10.1001/jama.2015.12 PMC4374744

[24] Prin M, Wunsch H. International comparisons of intensive care. Current Opinion in Critical Care. 2012;18(6):700–6. doi: 10.1097/mcc.0b013e32835914d5 22954664 PMC3551445

[25] Uğur M, Çevik E, Kumbasar AB, Keleş A. Damage control surgery at the Syrian border. Ulus Travma Acil Cerrahi Derg. 2016;22(6):535–9.

[26] van der Merwe E, Manchev V, Pillay B. Outcomes and complications of damage control laparotomy for trauma. S Afr J Surg. 2023;61(3):148–53.

[27] Kruger A, Nicol AJ, Navsaria PH. Damage control laparotomy for gunshot injuries to the abdomen. S Afr J Surg. 2022;60(1):43–7. 35851360

[28] Kisat M, Zafar SN, Hashmi ZG. Experience of damage control trauma surgery in a developing country. Int J Surg. 2016;28:71–6. 26889970 10.1016/j.ijsu.2016.02.042 PMC4826292

[29] Traynor MD Jr, Hernandez MC, Aho JM, Wise K, Kong V, Clarke D, et al. Damage Control Laparotomy: High-Volume Centers Display Similar Mortality Rates Despite Differences in Country Income Level. World J Surg. 2020;44(12):3993–8. doi: 10.1007/s00268-020-05718-5 32737559

[30] Kruger HIJ, Steyn E, Buitendag JJP, Klopper JH, Oosthuizen G. S Afr J Surg. 2026;64(1):48–52. 42157764 10.36303/SAJS.03262

[31] Abri MA, Snani SA, Almayahi J, Sharqi AA, Qadhi HA. The outcome of damage control surgery at Sultan Qaboos University Hospital. World J Surg Surgical Res. 2022;5:1428.

