# Supplementary material for: Outcomes of damage control laparotomy after trauma in low andmiddle-income countries: A systematic review and meta-analysis
Source: PLoS One. 2026 Jun 25;21(6):e0352357. doi: 10.1371/journal.pone.0352357 (PMC13298736; doi:10.1371/journal.pone.0352357)
Supplement: S1 Table — (DOCX) [file pone.0352357.s001.docx]

**Table S1. Detailed Search Strategy for Systematic Review**

**Systematic searching**

| *Database* | *Search strategy* | *Yields (number of records identified)* |
| --- | --- | --- |
| *Scopus* | "Damage control laparotomy" OR "DCL" OR "surgical intervention" OR "emergency surgery" AND trauma OR injury OR wound OR accident AND "low-income countries" OR "middle-income countries" OR "developing countries" OR "resource-limited settings" | *433* |
| *Science Direct* | damage control laparotomy AND trauma AND middle income countries | *311* |
| *Africa-Wide Information* | ("Damage control laparotomy" OR "DCL" OR "surgical intervention" OR "emergency surgery") AND (trauma OR injury OR wound OR accident) AND ("low-income countries" OR "middle-income countries" OR "developing countries" OR "resource-limited settings") | *99* |
| *CINAHL Ultimate* | same | *40* |
| *MEDLINE* | same | *278* |
| ***Pubmed*** | ***(("damage control surgery"[Title/Abstract] OR "damage control"[Title/Abstract] OR "abbreviated laparotomy"[Title/Abstract] OR "staged laparotomy"[Title/Abstract] OR "temporary abdominal closure"[Title/Abstract] OR DCS[Title/Abstract] OR "damage control resuscitation"[Title/Abstract] OR "damage control orthopedics"[Title/Abstract] OR "damage control orthopaedics"[Title/Abstract]) AND ("trauma"[MeSH Terms] OR "wounds and injuries"[MeSH Terms] OR trauma[Title/Abstract] OR injury[Title/Abstract] OR injuries[Title/Abstract] OR "polytrauma"[Title/Abstract] OR "multiple trauma"[Title/Abstract] OR "blunt trauma"[Title/Abstract] OR "penetrating trauma"[Title/Abstract]) AND ("mortality"[MeSH Terms] OR "morbidity"[MeSH Terms] OR mortality[Title/Abstract] OR morbidity[Title/Abstract] OR death[Title/Abstract] OR deaths[Title/Abstract] OR survival[Title/Abstract] OR "survival rate"[Title/Abstract] OR complication*[Title/Abstract] OR outcome*[Title/Abstract] OR "adverse events"[Title/Abstract]) AND ("developing countries"[MeSH Terms] OR "low income country"[Title/Abstract] OR "low income countries"[Title/Abstract] OR "middle income country"[Title/Abstract] OR "middle income countries"[Title/Abstract] OR LMIC[Title/Abstract] OR LMICs[Title/Abstract] OR "low-resource"[Title/Abstract] OR "resource-limited"[Title/Abstract] OR "resource-poor"[Title/Abstract] OR Africa[Title/Abstract] OR Asia[Title/Abstract] OR "Latin America"[Title/Abstract] OR "South America"[Title/Abstract] OR "sub-Saharan"[Title/Abstract])) AND (2004:2024[pdat])  2004-2026*** | ***38*** |
