# Supplementary material for: Outcomes of damage control laparotomy after trauma in low andmiddle-income countries: A systematic review and meta-analysis
Source: PLoS One. 2026 Jun 25;21(6):e0352357. doi: 10.1371/journal.pone.0352357 (PMC13298736; doi:10.1371/journal.pone.0352357)
Supplement: S1 File — (DOCX) [file pone.0352357.s002.docx]

| **Section and Topic** | **Item #** | **Checklist item** | **Location where item is reported** |
| --- | --- | --- | --- |
| **TITLE** | | |  |
| Title | 1 | Identify the report as a systematic review. | Title page: "...A Systematic Review and Meta-Analysis" |
| **ABSTRACT** | | |  |
| Abstract | 2 | See the PRISMA 2020 for Abstracts checklist. | Abstract (structured: Background, Methods, Results, Conclusions) |
| **INTRODUCTION** | | |  |
| Rationale | 3 | Describe the rationale for the review in the context of existing knowledge. | Introduction, paragraphs 1–4 |
| Objectives | 4 | Provide an explicit statement of the objective(s) or question(s) the review addresses. | Introduction, final paragraph |
| **METHODS** | | |  |
| Eligibility criteria | 5 | Specify the inclusion and exclusion criteria for the review and how studies were grouped for the syntheses. | Methods – Eligibility Criteria; grouping described in Methods – Statistical Analysis and Results – Subgroup Analysis |
| Information sources | 6 | Specify all databases, registers, websites, organisations, reference lists and other sources searched or consulted to identify studies. Specify the date when each source was last searched or consulted. | Methods – Search Strategy (PubMed/MEDLINE, Scopus, Google Scholar, Cochrane Library, AJOL; 01 Jan 2004 – 28 Feb 2026) |
| Search strategy | 7 | Present the full search strategies for all databases, registers and websites, including any filters and limits used. | PARTIAL — conceptual domains described in Methods – Search Strategy; full database-specific Boolean strings not presented. See Supplementary Appendix 1 . |
| Selection process | 8 | Specify the methods used to decide whether a study met the inclusion criteria of the review, including how many reviewers screened each record and each report retrieved, whether they worked independently, and if applicable, details of automation tools used in the process. | Methods – Study Selection and Data Extraction (two independent reviewers, consensus resolution, no automation tools) |
| Data collection process | 9 | Specify the methods used to collect data from reports, including how many reviewers collected data from each report, whether they worked independently, any processes for obtaining or confirming data from study investigators, and if applicable, details of automation tools used in the process. | Methods – Study Selection and Data Extraction (standardised extraction form, two reviewers) |
| Data items | 10a | List and define all outcomes for which data were sought. Specify whether all results that were compatible with each outcome domain in each study were sought (e.g. for all measures, time points, analyses), and if not, the methods used to decide which results to collect. | Methods – Study Selection and Data Extraction; primary outcome (mortality) and secondary outcomes (complications, LOS, cost) defined throughout Results |
|  | 10b | List and define all other variables for which data were sought (e.g. participant and intervention characteristics, funding sources). Describe any assumptions made about any missing or unclear information. | PARTIAL — variables listed in Methods – Study Selection and Data Extraction; assumptions about missing data not explicitly stated. See addition in Part 5. |
| Study risk of bias assessment | 11 | Specify the methods used to assess risk of bias in the included studies, including details of the tool(s) used, how many reviewers assessed each study and whether they worked independently, and if applicable, details of automation tools used in the process. | Methods – Risk of Bias Assessment (modified Newcastle-Ottawa Scale) |
| Effect measures | 12 | Specify for each outcome the effect measure(s) (e.g. risk ratio, mean difference) used in the synthesis or presentation of results. | Methods – Statistical Analysis (logit-transformed proportions, back-transformed to pooled mortality proportion with 95% CI) |
| Synthesis methods | 13a | Describe the processes used to decide which studies were eligible for each synthesis (e.g. tabulating the study intervention characteristics and comparing against the planned groups for each synthesis (item #5)). | Methods – Eligibility Criteria, Duplicate Population Identification, and Statistical Analysis |
|  | 13b | Describe any methods required to prepare the data for presentation or synthesis, such as handling of missing summary statistics, or data conversions. | Methods – Statistical Analysis (logit transformation, 0.5 continuity correction) |
|  | 13c | Describe any methods used to tabulate or visually display results of individual studies and syntheses. | Methods – Statistical Analysis; Results – Figure 1 (PRISMA flow), Figure 2 (forest plot), Figure 3 (subgroup forest plot), Figure 4 (funnel plot), Tables 1–2 |
|  | 13d | Describe any methods used to synthesize results and provide a rationale for the choice(s). If meta-analysis was performed, describe the model(s), method(s) to identify the presence and extent of statistical heterogeneity, and software package(s) used. | Methods – Statistical Analysis (DerSimonian-Laird random- effects model; I2, τ2, Cochran Q, H2; Stata v18) |
|  | 13e | Describe any methods used to explore possible causes of heterogeneity among study results (e.g. subgroup analysis, meta-regression). | Methods – Statistical Analysis (pre-specified subgroup analysis by geographic region: South Africa vs. other LMICs) |
|  | 13f | Describe any sensitivity analyses conducted to assess robustness of the synthesized results. | PARTIAL — exclusion of a non- DCS hepatic trauma cohort described narratively in Results; formal leave-one-out sensitivity analysis not reported. See addition in Part 5. |
| Reporting bias assessment | 14 | Describe any methods used to assess risk of bias due to missing results in a synthesis (arising from reporting biases). | Methods – Statistical Analysis (funnel plot inspection; Egger's regression test, p = 0.299) |
| Certainty assessment | 15 | Describe any methods used to assess certainty (or confidence) in the body of evidence for an outcome. | NOT REPORTED — GRADE assessment not performed. See addition in Part 5. |
| **RESULTS** | | |  |
| Study selection | 16a | Describe the results of the search and selection process, from the number of records identified in the search to the number of studies included in the review, ideally using a flow diagram. | Results – Study Selection; Figure 1 (PRISMA flow diagram) |
|  | 16b | Cite studies that might appear to meet the inclusion criteria, but which were excluded, and explain why they were excluded. | Results – Study Selection (reasons for exclusion listed in Figure 1); Methods – Duplicate Population Identification (Weale et al. TSACO 2019 included) |
| Study characteristics | 17 | Cite each included study and present its characteristics. | Results – Study Characteristics; Table 1 |
| Risk of bias in studies | 18 | Present assessments of risk of bias for each included study. | PARTIAL — tool described in Methods; study-level NOS scores not presented in table/figure form. See Table 2 in Part 3 of this document. |
| Results of individual studies | 19 | For all outcomes, present, for each study: (a) summary statistics for each group (where appropriate) and (b) an effect estimate and its precision (e.g. confidence/credible interval), ideally using structured tables or plots. | Table 1 (individual study mortality rates); Figure 2 (forest plot with study-level estimates and 95% CIs) |
| Results of syntheses | 20a | For each synthesis, briefly summarise the characteristics and risk of bias among contributing studies. | Results – Study Characteristics; Discussion – Strengths and Limitations |
|  | 20b | Present results of all statistical syntheses conducted. If meta-analysis was done, present for each the summary estimate and its precision (e.g. confidence/credible interval) and measures of statistical heterogeneity. If comparing groups, describe the direction of the effect. | Results – Primary Outcome: Mortality; Table 2 (pooled mortality 37.77%, 95% CI 31.38– 44.62%; I2 = 74.46%) |
|  | 20c | Present results of all investigations of possible causes of heterogeneity among study results. | Results – Subgroup Analysis by Region (SA: 31.61%, I2=0%; other LMICs: 51.57%, I2=48%) |
|  | 20d | Present results of all sensitivity analyses conducted to assess the robustness of the synthesized results. | PARTIAL — exclusion of non- DCS cohort described narratively; formal sensitivity analysis results not tabulated. See addition in Part 5. |
| Reporting biases | 21 | Present assessments of risk of bias due to missing results (arising from reporting biases) for each synthesis assessed. | Results – Publication Bias; Figure 4 (funnel plot); Egger's test p = 0.299; mild asymmetry interpreted as likely due to true heterogeneity |
| Certainty of evidence | 22 | Present assessments of certainty (or confidence) in the body of evidence for each outcome assessed. | NOT REPORTED — GRADE assessment not performed. See Part 4 of this document. |
| **DISCUSSION** | | |  |
| Discussion | 23a | Provide a general interpretation of the results in the context of other evidence. | Discussion – Summary of Main Findings; Comparison with High- Income Country Data |
|  | 23b | Discuss any limitations of the evidence included in the review. | Discussion – Strengths and Limitations |
|  | 23c | Discuss any limitations of the review processes used. | Discussion – Strengths and Limitations |
|  | 23d | Discuss implications of the results for practice, policy, and future research. | Discussion – Clinical Implications; Conclusions |
| **OTHER INFORMATION** | | |  |
| Registration and protocol | 24a | Provide registration information for the review, including register name and registration number, or state that the review was not registered. | Methods, first paragraph: PROSPERO CRD420251037759 |
|  | 24b | Indicate where the review protocol can be accessed, or state that a protocol was not prepared. | PARTIAL — registration stated but access location not provided. Add: "The protocol is accessible via the PROSPERO database at https://www.crd.york.ac.uk/ prospero/display_record.php? RecordID=1037759" |
|  | 24c | Describe and explain any amendments to information provided at registration or in the protocol. | No amendments were made to the registered protocol after registration |
| Support | 25 | Describe sources of financial or non-financial support for the review, and the role of the funders or sponsors in the review. | Funding section: "This research received no specific grant from any funding agency in the public, commercial, or not-for-profit sectors." |
| Competing interests | 26 | Declare any competing interests of review authors. | Competing interests section: "The authors declare that they have no competing interests." |
| Availability of data, code and other materials | 27 | Report which of the following are publicly available and where they can be found: template data collection forms; data extracted from included studies; data used for all analyses; analytic code; any other materials used in the review. | PARTIAL — Availability of Data and Materials section states data available on request. Extraction forms, analytic code, and supplementary materials not explicitly addressed. |

*From:*  Page MJ, McKenzie JE, Bossuyt PM, Boutron I, Hoffmann TC, Mulrow CD, et al. The PRISMA 2020 statement: an updated guideline for reporting systematic reviews. BMJ 2021;372:n71. doi: 10.1136/bmj.n71. This work is licensed under CC BY 4.0. To view a copy of this license, visit <https://creativecommons.org/licenses/by/4.0/>
