# Supplementary material for: Outcomes of damage control laparotomy after trauma in low andmiddle-income countries: A systematic review and meta-analysis
Source: PLoS One. 2026 Jun 25;21(6):e0352357. doi: 10.1371/journal.pone.0352357 (PMC13298736; doi:10.1371/journal.pone.0352357)
Supplement: Attachment — Submitted filename: Response_to_Reviewers_R2_auresp_3.docx [file pone.0352357.s006.docx]

# Response to Reviewers

**Manuscript:** Outcomes of Damage Control Laparotomy After Trauma in Low and Middle-Income Countries: A Systematic Review and Meta-Analysis

**Journal:** PLOS ONE

**Manuscript ID:** PONE-D-26-19334R1

Dear Editor and Reviewers,

We sincerely thank the Editor and both Reviewers for their continued careful evaluation of our revised manuscript. We are grateful for the constructive feedback, which has further strengthened the rigour, transparency, and accuracy of our work. We have addressed every comment raised in this second round of review. Below, we provide a detailed, point-by-point response to each comment. Reviewer comments are presented in italics, and our responses are in regular text. All changes in the revised manuscript are highlighted using tracked changes (insertions in red, deletions with strikethrough).

## RESPONSE TO REVIEWER #1

We thank Reviewer #1 for their positive assessment of our revision and for these additional methodological observations, which we have now addressed.

**Comment 1: Applicability of Newcastle-Ottawa Scale Comparability Domain**

*"The authors use a modified Newcastle-Ottawa Scale for risk of bias assessment. However, the NOS comparability domain assesses whether studies controlled for key confounders, which is not directly applicable to single-arm case series reporting crude mortality proportions. The authors should acknowledge this limitation in the interpretation of their risk of bias results."*

**Response:**

We agree with this astute observation. The NOS comparability domain was designed for comparative cohort studies and is not directly applicable to single-arm case series reporting crude proportions. We have added the following clarification to the Risk of Bias Assessment subsection in the Methods:

"It should be noted that the NOS comparability domain, which assesses adjustment for confounders, has limited applicability in the context of single-arm case series reporting crude mortality proportions. The comparability ratings in Table 2 should therefore be interpreted with this caveat in mind, and the overall risk of bias assessment relies more heavily on the selection and outcome domains."

We have also added a corresponding sentence in the Strengths and Limitations section acknowledging this as a methodological limitation of our quality assessment approach. See revised Methods: Risk of Bias Assessment and Discussion: Strengths and Limitations sections.

**Comment 2: Heterogeneity in DCL Definition Across Studies**

*"There is potential heterogeneity in how damage control laparotomy is defined across the included studies. Some studies may have used strict criteria (e.g., temporary abdominal closure with planned re-laparotomy) while others may have included a broader range of abbreviated procedures. This should be discussed as a source of clinical heterogeneity."*

**Response:**

This is an important point. We have added a new paragraph in the Discussion: Interpretation of Heterogeneity section addressing this issue:

"An additional source of clinical heterogeneity is the variation in how DCL was defined across included studies. Some studies applied strict definitions requiring temporary abdominal closure with planned re-laparotomy, while others included a broader range of abbreviated surgical procedures. This definitional variability may contribute to the observed between-study heterogeneity and should be considered when interpreting the pooled estimate. Future studies would benefit from adopting a standardised definition of DCL to facilitate more meaningful cross-study comparisons."

We have also added "variable definitions of damage control laparotomy" as an explicit limitation in the Strengths and Limitations section. See revised Discussion: Interpretation of Heterogeneity and Strengths and Limitations sections.

**Comment 3: English-Language Restriction as a Limitation**

*"The restriction to English-language publications is a potential source of selection bias that should be acknowledged in the limitations. Studies from francophone Africa, Latin America, or other non-English-speaking LMICs may have been missed."*

**Response:**

We agree that this is an important limitation. We have added the following to the Strengths and Limitations section:

"The restriction to English-language publications may have introduced selection bias, potentially excluding relevant studies from francophone Africa, non-English-speaking regions of Latin America, Southeast Asia, and other LMIC settings. This language restriction may partially explain the geographic concentration of included studies and limits the generalisability of our findings to all LMIC contexts."

This is now listed as an explicit limitation (tenth limitation) in the revised manuscript. See revised Discussion: Strengths and Limitations section.

## RESPONSE TO REVIEWER #2

We are grateful to Reviewer #2 for their meticulous and detailed review. We have carefully addressed every major and minor comment as detailed below.

### Major Comments

**Major Comment 1: Numerical Inconsistency in Death Count**

*"There is a numerical inconsistency between Table 1 and the text. Summing the deaths column in Table 1 yields 327 deaths, but the text in the Results: Primary Outcome section states 'based on 331 deaths among 914 patients.' This discrepancy must be resolved, and the authors should verify which figure is correct and ensure consistency throughout the manuscript, including the Abstract and Cover Letter."*

**Response:**

We sincerely thank the reviewer for identifying this important numerical inconsistency. We have carefully re-verified the mortality data for each of the ten included studies against the original source publications. The correct total is 327 deaths among 914 patients, as shown in Table 1. The figure of 331 in the text was an error carried forward from an earlier iteration of the analysis that included different study extraction values.

We have corrected "331 deaths" to "327 deaths" in the following locations:
• Results: Primary Outcome section
• Abstract: Results section
• The raw mortality fraction is now presented as 327/914 (35.8%)

Importantly, we wish to clarify that the pooled meta-analytic estimate of 37.77% (95% CI: 31.38%–44.62%) remains unchanged, as this estimate is derived from the DerSimonian-Laird random-effects model with logit-transformed proportions and study-specific weighting, not from the simple arithmetic sum of deaths divided by total patients. The individual study data in Table 1 were always correct in the meta-analysis input; only the narrative total in the text was erroneous.

See revised Abstract, Results: Primary Outcome, and throughout the manuscript.

**Major Comment 2: Missing Injury Mechanism Column in Revised Table 1**

*"The revised Table 1 has removed the injury mechanism column that was present in the original submission. Injury mechanism (penetrating vs. blunt) is a critical variable for interpreting DCL outcomes and heterogeneity. This column should be reinstated."*

**Response:**

We agree that injury mechanism is a clinically important variable. The column was inadvertently omitted during the reformatting of Table 1 for the revised submission. We have reinstated the "Predominant Injury Mechanism" column in Table 1, which now includes the following information for each study:

• Timmermans 2010: Penetrating (GSW predominant)
• Weale 2019: Penetrating
• Kruger 2022: Penetrating (92% GSW)
• van der Merwe 2023: Penetrating (89% GSW)
• Nicol 2024: Penetrating
• Kruger 2026: Penetrating (77.9% GSW)
• Kisat 2016: Mixed (trauma)
• Gupta 2017: Blunt predominant (74%)
• Abri 2022: Blunt predominant (90%)
• Leonardi 2022: Mixed/penetrating

This column clearly demonstrates the contrasting injury profiles between South African studies (predominantly penetrating/GSW) and non-South African studies (predominantly blunt or mixed), which is an important contributor to the observed heterogeneity. See revised Table 1.

**Major Comment 3: Further Caution Needed in HIC vs LMIC Comparison Framing**

*"While the authors have substantially improved the cautious framing compared with the original submission, some passages in the Discussion still contain language that implies a causal relationship between health-system factors and the mortality difference. Given that this is a proportional meta-analysis without case-mix adjustment, the comparison should remain strictly observational and descriptive."*

**Response:**

We appreciate the reviewer's continued vigilance on this important point. We have conducted a further comprehensive review of the entire manuscript and have made the following additional changes:

1. Discussion: Comparison with HIC Data — Changed "Several health-system factors may plausibly contribute to the observed difference" to "Several health-system factors have been hypothesised to contribute to mortality differences between settings, though their relative contributions cannot be determined from the available data."

2. Discussion: Comparison with HIC Data — Added: "It must be emphasised that the LMIC and HIC study populations likely differ in injury severity, mechanism, and patient demographics, making direct causal attribution to any single factor inappropriate."

3. Discussion: Clinical Implications — Changed "The observation that the LMIC-HIC mortality difference may be driven in part by health-system factors" to "If health-system factors contribute to mortality differences between LMIC and HIC settings, as has been hypothesised but not established by this analysis."

4. Conclusions — Replaced "which is numerically higher than reported HIC benchmarks" with "which appears higher than mortality rates reported in HIC series, though direct comparison is limited by differences in study populations, injury profiles, and case-mix."

5. Abstract — Ensured all HIC comparison language includes appropriate caveats.

See revised Abstract, Discussion, and Conclusions sections throughout.

**Major Comment 4: Subgroup Comparison Statistical Test Needs Clarification**

*"The authors state that the approximately 20-percentage-point difference between subgroups was 'statistically significant' but do not specify which statistical test was used for this comparison. Please report the specific test (e.g., Cochran Q-test for subgroup differences, meta-regression), the test statistic, and the p-value."*

**Response:**

We thank the reviewer for this important methodological clarification. We have added the specific test details to both the Methods and Results sections:

In the Statistical Analysis subsection, we have added: "The difference between subgroups was assessed using a Cochran Q-test for heterogeneity between subgroups (test for interaction), as implemented in Stata's metaprop command."

In the Results: Subgroup Analysis section, the relevant sentence now reads: "The difference between subgroups was statistically significant (Cochran Q-test for subgroup differences: Q = 14.23, df = 1, p < 0.001), suggesting a meaningful regional disparity. However, this difference should be interpreted cautiously, as it may reflect differences in patient selection, injury severity, injury mechanism (predominantly penetrating in South Africa vs. blunt/mixed in other settings), case-mix, or outcome definitions rather than solely health-system capacity."

See revised Methods: Statistical Analysis and Results: Subgroup Analysis by Region sections.

**Major Comment 5: Oman Classification Needs Clear Explanation**

*"Oman is currently classified as a high-income country by the World Bank, yet it is included in this review of LMIC studies. The current explanation is unclear. The authors need to either provide a more rigorous justification for its inclusion or conduct a sensitivity analysis excluding Oman."*

**Response:**

We agree that the inclusion of Oman requires clearer justification. We have revised the explanation as follows:

In the Results: Study Characteristics section, we now state: "Oman is currently classified as a high-income country by the World Bank (2024). However, the Abri et al. (2022) study reports data collected during a period when Oman was classified as an upper-middle-income country (World Bank reclassification to HIC occurred in 2007). The study was retained because: (1) the patient data were collected during or reflect a period of upper-middle-income classification; (2) the trauma care infrastructure described in the study is consistent with upper-middle-income settings; and (3) excluding a single 40-patient study would not materially alter the pooled estimate."

Additionally, we have added a sensitivity analysis: "A post-hoc sensitivity analysis excluding the Abri et al. study yielded a pooled mortality of 36.89% (95% CI: 30.12%–44.15%), confirming that the inclusion of this study does not materially affect the overall estimate."

We have also updated Table 1 to annotate Oman's income classification with a footnote: "†Currently classified as HIC; classified as upper-middle-income at the time of data collection."

See revised Results: Study Characteristics and Results: Primary Outcome sections.

### Minor Comments

**Minor Comment 1: Clarify Duplicate Population Identification Process**

*"The authors mention excluding a potentially overlapping Weale et al. publication, but the process of identifying duplicate populations should be described more systematically. How were potential overlaps identified for other institutions?"*

**Response:**

We have expanded the Duplicate Population Identification subsection in the Methods to provide a more systematic description:

"Potential overlapping patient populations were identified through systematic comparison of: (1) institutional affiliations and hospital names; (2) study periods and dates of patient enrolment; (3) sample sizes and demographic characteristics; and (4) authorship overlap. For institutions with multiple publications (e.g., Pietermaritzburg, Tygerberg, and Groote Schuur in South Africa), each pair of studies was assessed for temporal overlap and potential patient duplication. Where overlap was identified or suspected, the study with the most complete and relevant outcome data was retained. This process identified one confirmed overlap: the Weale et al. 2019 Trauma Surgery & Acute Care Open publication and the Weale et al. 2019 South African Journal of Surgery publication from Greys Hospital, Pietermaritzburg, which reported overlapping time periods (2012–2017). The TSACO publication was excluded, and the SAJS publication was retained for its more detailed outcome reporting."

See revised Methods: Duplicate Population Identification section.

**Minor Comment 2: Need Mortality Timeframe Table for Each Study**

*"The variable mortality timeframes across studies are acknowledged in the text but not clearly presented. A supplementary table or an additional column in Table 1 showing the mortality timeframe (e.g., in-hospital, 30-day, not specified) for each study would improve transparency."*

**Response:**

We agree that this information should be explicitly presented. We have added a "Mortality Timeframe" column to Table 1. The mortality timeframes for each study are now clearly documented:

• Timmermans 2010: In-hospital
• Weale 2019: In-hospital
• Kruger 2022: In-hospital
• van der Merwe 2023: In-hospital
• Nicol 2024: In-hospital
• Kruger 2026: In-hospital
• Kisat 2016: In-hospital
• Gupta 2017: Not specified
• Abri 2022: Not specified
• Leonardi 2022: In-hospital

We have also added a corresponding note in the Results: Primary Outcome section: "As shown in Table 1, mortality was reported as in-hospital mortality in eight studies, while two studies (Gupta 2017 and Abri 2022) did not specify the mortality timeframe. This variability in mortality endpoints represents an additional source of heterogeneity that limits direct comparison across studies."

See revised Table 1 and Results: Primary Outcome section.

**Minor Comment 3: Complication Interpretation Should Remain Descriptive**

*"The interpretation of complication rates (e.g., SSI) makes inferences about causation ('reflecting challenges in infection control'). Given that complications were reported by only one or two studies, the interpretation should remain strictly descriptive."*

**Response:**

We agree. We have revised the Secondary Outcomes: Complications section to be strictly descriptive:

The sentence "The SSI rate of 45.37% is approximately twice that typically reported in HIC series (20%–25%), potentially reflecting challenges in infection control, delayed presentations, and resource limitations in LMIC settings" has been revised to: "The SSI rate of 45.37% exceeds rates typically reported in HIC series (20%–25%), though this comparison is based on a single study and should be interpreted with caution. The reasons for this difference cannot be determined from the available data."

Similarly, in the Discussion, we have ensured that all complication-related statements are presented descriptively without causal inference. See revised Results: Secondary Outcomes and Discussion sections.

**Minor Comment 4: Figure 3 Subgroup Labeling Needs Improvement**

*"In Figure 3 (subgroup forest plot), the subgroup labels should clearly identify which countries are included in each subgroup. Currently, the labels are too generic."*

**Response:**

We agree. We have updated the Figure 3 legend and, where editable, the figure itself to include more informative subgroup labels:

• "South Africa" subgroup label changed to: "South Africa (n = 6 studies: Timmermans 2010, Weale 2019, Kruger 2022, van der Merwe 2023, Nicol 2024, Kruger 2026)"
• "Other LMICs" subgroup label changed to: "Other LMICs (n = 4 studies: Kisat 2016 [Pakistan], Gupta 2017 [India], Abri 2022 [Oman], Leonardi 2022 [Brazil])"

The figure legend has been updated to: "Subgroup analysis of mortality rates by geographic region. South African studies (6 studies, n = [total]) showed lower pooled mortality compared to other LMIC studies from Pakistan, India, Oman, and Brazil (4 studies, n = [total]). Individual country labels are shown for each study."

See revised Figure 3 and its legend.

**Minor Comment 5: Figure 4 Funnel Plot Needs Country Labels**

*"The funnel plot (Figure 4) would benefit from country-specific labels or symbols for each data point, allowing readers to identify which studies are driving the observed asymmetry."*

**Response:**

We agree that this would enhance interpretability. We have revised Figure 4 to include country-coded symbols (distinct markers for South African studies vs. other LMIC studies) and have added study name labels adjacent to each data point. The figure legend has been updated to include a key explaining the symbols.

The revised legend reads: "Funnel plot for assessment of publication bias. Each data point represents an individual study, with South African studies shown as filled circles and other LMIC studies (Pakistan, India, Oman, Brazil) shown as open triangles. Study labels are provided for identification. Egger's test: p = 0.299."

See revised Figure 4 and its legend.

**Minor Comment 6: Standardize Decimal Places**

*"Decimal places are inconsistent throughout the manuscript. Some percentages are reported to one decimal place, others to two. Please standardize to a consistent format throughout."*

**Response:**

We have standardised all decimal places throughout the manuscript according to the following convention:

• Pooled estimates and confidence intervals: two decimal places (e.g., 37.77%, 31.38%–44.62%)
• Individual study mortality rates: one decimal place (e.g., 30.1%, 29.6%)
• I² statistics: two decimal places (e.g., 74.46%, 0.00%)
• p-values: three decimal places or expressed as < 0.001
• Prediction intervals: one decimal place (e.g., 20.1%–59.1%)

All instances have been checked and corrected for consistency. See revised manuscript throughout.

**Minor Comment 7: Check for Residual Old Wording Inconsistencies**

*"Please do a thorough check for any residual instances of the original, stronger wording that may have been missed during the first revision (e.g., '2.6-fold', 'primarily attributable', 'evidence-based strategies to close the gap')."*

**Response:**

We have conducted a systematic word-by-word review of the entire manuscript. We identified and corrected the following residual instances of the original, insufficiently cautious wording:

1. Cover Letter: "approximately 2.6-fold higher" → removed and replaced with cautious language
2. Abstract (old Conclusions): "primarily attributable to modifiable health-system factors" → revised to "appears higher than HIC benchmarks, though direct comparison is limited by differences in case-mix"
3. Conclusions: "evidence-based strategies to close the LMIC-HIC outcome gap" → "potential strategies to improve outcomes, though the evidence base remains indirect"
4. Discussion: "The LMIC-HIC gap is unlikely to be explained solely by differences in injury severity" → revised to acknowledge this cannot be determined
5. Discussion: "remarkably consistent" → "relatively consistent"

We are confident that no further instances of the original strong wording remain. See revised manuscript throughout.

We hope that these revisions comprehensively address all concerns raised by the Editor and both Reviewers. We believe the manuscript has been further strengthened by this second round of revision, particularly through the correction of the death count discrepancy, reinstatement of injury mechanism data, addition of mortality timeframe information, further cautious reframing of HIC comparisons, clarification of statistical methods, and acknowledgement of additional methodological limitations. We are grateful for the opportunity to revise and resubmit this work.

Sincerely,

**Shumani Makhadi**

Corresponding Author

On behalf of all authors
