# Supplementary material for: Outcomes of damage control laparotomy after trauma in low andmiddle-income countries: A systematic review and meta-analysis
Source: PLoS One. 2026 Jun 25;21(6):e0352357. doi: 10.1371/journal.pone.0352357 (PMC13298736; doi:10.1371/journal.pone.0352357)
Supplement: Attachment — Submitted filename: Response_to_Reviewers DCL.docx [file pone.0352357.s004.docx]

**Response to Reviewers**

*Manuscript: Outcomes of Damage Control Laparotomy After Trauma in Low and Middle-Income Countries: A Systematic Review and Meta-Analysis*

Journal: PLOS ONE

Manuscript ID: PONE-D-26-19334

Dear Editor and Reviewers,

We sincerely thank the Editor and both Reviewers for their thorough and constructive evaluation of our manuscript. Their feedback has been invaluable in strengthening the methodological rigour, transparency, and balance of our work. We have carefully considered each comment and made substantial revisions to the manuscript accordingly. Below, we provide a detailed, point-by-point response to each comment. Reviewer comments are presented in italics, and our responses are in regular text. All changes are highlighted in the tracked-changes version of the manuscript.

# RESPONSE TO EDITOR'S COMMENTS

**Editor's Point 1:**

*"Please ensure that your manuscript meets PLOS ONE's style requirements, including those for file naming."*

**Response:** We have reviewed the PLOS ONE style requirements and reformatted the manuscript accordingly. File names have been updated to comply with journal guidelines. All sections follow the required formatting structure.

**Editor's Point 2:**

*"Please provide a complete Data Availability Statement in the submission form, indicating where the data may be found."*

**Response:** We have updated the Data Availability Statement to read: "All data underlying the findings of this systematic review are available within the article and its Supplementary Material. The extracted data from included studies are presented in Table 1. The complete search strategy is provided in Supplementary Material S1." This statement has been made consistent between the submission form and the manuscript (see revised Data Availability Statement section).

**Editor's Point 3:**

*"Please include captions for your Supporting Information files at the end of your manuscript, and update any in-text citations to match accordingly."*

**Response:** We have added proper captions for all Supplementary Material at the end of the manuscript, including: "Supplementary Material S1. Detailed search strategy for each database" and "Table S1. Complete search strategy for PubMed/MEDLINE, Scopus, Google Scholar, Cochrane Library, and African Journals Online (AJOL), including search terms, Boolean operators, filters, and number of records retrieved per database." In-text citations to supplementary material have been updated throughout (see Methods: Search Strategy section).

**Editor's Point 4:**

*"Please include your tables as part of your main manuscript and remove the individual files."*

**Response:** Tables 1 and 2 have been incorporated into the main manuscript body. Table 1 (Characteristics of included studies) and Table 2 (Risk of bias assessment) now appear within the Results: Study Characteristics section.

**Editor's Point 5:**

*"Please review your reference list to ensure that it is complete and correct."*

**Response:** The reference list has been thoroughly reviewed. All 31 references have been verified for completeness and accuracy, including author names, titles, journal names, publication years, volume/issue numbers, and page ranges.

**Editor's Point 6:**

*"Please ensure that the abstract in the manuscript matches the abstract in the submission form."*

**Response:** We have ensured that the abstract is identical between the manuscript and the submission form. The revised abstract now includes the prediction interval (95% PI: 20.1%–59.1%), acknowledgement of South African predominance in the dataset, and appropriately cautious conclusions. Both versions are now consistent.

**Editor's Point 7:**

*"Please include your ethics statement in the Methods section of your manuscript."*

**Response:** The ethics statement has been moved from the end-matter declarations to the Methods section, immediately after the protocol registration statement and before the Search Strategy subsection. The statement reads: "This study is a systematic review and meta-analysis of previously published studies and did not involve direct interaction with human participants. Ethical approval was therefore not required." The duplicate statement has been removed from the end-matter.

**Editor's Point 8:**

*"Please upload a copy of your PRISMA checklist as Supporting Information."*

**Response:** We have uploaded the completed PRISMA 2020 checklist as Supporting Information (Supplementary Material S2) with the revised submission. Each item is cross-referenced to the relevant manuscript page number or section.

# RESPONSE TO REVIEWER #1

We thank Reviewer #1 for their careful and expert evaluation of our manuscript. Their methodological suggestions have substantially improved the rigour and transparency of our analysis.

## Reviewer #1 — Methods

**Comment M1:**

*"The search strategy should be described in more detail. Please provide the complete search strings used for each database, or include these as supplementary material."*

**Response:** We agree that the search strategy required greater transparency. We have: (1) added a reference to the detailed search strategy in Supplementary Material S1 (Table S1), which provides the complete search strings, Boolean operators, filters, and number of records retrieved for each of the five databases; and (2) expanded the Search Strategy subsection in the Methods to include more detail on the search terms and Boolean logic used. See revised Methods: Search Strategy section and Supplementary Material S1.

**Comment M2:**

*"The statistical methods section should include prediction intervals to complement the confidence intervals, given the high heterogeneity observed."*

**Response:** We fully agree with this important recommendation. We have: (1) added 95% prediction intervals to all pooled estimates in the Results section (overall pooled mortality: 95% PI: 20.1%–59.1%); (2) added a new paragraph in the Statistical Analysis subsection explaining the calculation and interpretation of prediction intervals; (3) included prediction intervals in the Abstract and Conclusions; and (4) discussed the prediction interval in the Discussion section. This provides a more complete picture of the expected range of outcomes. See revised Statistical Analysis, Results: Primary Outcome, Abstract, and Conclusions sections.

**Comment M3:**

*"Please clarify the definition of LMIC used in this review and specify which World Bank classification year was applied."*

**Response:** We have added a new paragraph in the Methods: Search Strategy section explicitly defining LMICs according to the World Bank income classification system (2024 fiscal year), including the GNI per capita thresholds for each income category. We have clarified that 'LMICs' encompasses low-income, lower-middle-income, and upper-middle-income countries, consistent with the global surgery literature convention. See revised Methods: Search Strategy section.

## Reviewer #1 — Results

**Comment R1:**

*"The claim of a '2.6-fold increase' in mortality compared with HICs should be qualified, as this is a comparison of crude proportions from different study populations without case-mix adjustment."*

**Response:** We agree that this comparison was insufficiently qualified. We have: (1) removed the specific '2.6-fold' language throughout the manuscript; (2) replaced it with more cautious phrasing such as 'numerically higher than reported HIC benchmarks' and 'direct comparison is limited by differences in study design, patient selection, and case-mix'; (3) added explicit caveats about the absence of case-mix adjustment whenever LMIC-HIC comparisons are made. See revised Results: Primary Outcome, Discussion: Summary of Main Findings, and Conclusions sections.

**Comment R2:**

*"Please acknowledge the South African predominance in the dataset more prominently and discuss how this affects the generalisability of the pooled estimates."*

**Response:** We have substantially strengthened the acknowledgement of South African predominance throughout the manuscript. Specifically: (1) added a new paragraph in Results: Study Characteristics explicitly noting that South Africa contributed 6/10 studies and 77.0% of the total patient population; (2) added this information to the Abstract; (3) added a dedicated paragraph in the Discussion: Summary of Main Findings section discussing how this predominance limits generalisability; (4) expanded the Limitations section to specifically address the implications of South African predominance. See revised Results, Abstract, Discussion, and Limitations sections.

## Reviewer #1 — Discussion

**Comment D1:**

*"The conclusions and clinical implications are overly strong given the limitations of the evidence base. Please tone down the language and add appropriate hedging."*

**Response:** We agree and have substantially revised the language throughout the Discussion and Conclusions. Key changes include: (1) replacing definitive statements ('is driven by') with hedged language ('may be driven in part by', 'potentially attributable to'); (2) adding 'if confirmed by future studies with appropriate case-mix adjustment' before clinical recommendations; (3) adding 'These recommendations are offered tentatively and will require validation in prospective studies'; (4) rewriting the Conclusions to explicitly state the limitations of comparison. See revised Discussion: Clinical Implications, Discussion: Summary of Main Findings, and Conclusions sections.

**Comment D2:**

*"The limitations section should be expanded to include the inherent limitation of proportional meta-analysis pooling crude mortality proportions without case-mix adjustment."*

**Response:** We have substantially expanded the Limitations section. We have added a specific limitation (now listed as the fifth limitation) addressing the proportional meta-analysis approach: 'the proportional meta-analysis approach pools crude mortality proportions without adjustment for case-mix, injury severity, or patient demographics. This means that differences in the pooled mortality between LMIC and HIC settings cannot be attributed solely to health-system factors; unmeasured confounders related to patient selection and injury characteristics may contribute substantially to the observed difference.' We also added this caveat in the Statistical Analysis section and Discussion: Summary of Main Findings. See revised Methods: Statistical Analysis, Discussion: Strengths and Limitations, and Discussion: Summary of Main Findings sections.

## Reviewer #1 — Conclusions

**Comment C1:**

*"The conclusion should include the prediction interval and should be more cautious in its claims about the LMIC-HIC mortality gap."*

**Response:** The Conclusions section has been substantially rewritten. It now: (1) includes the 95% prediction interval (20.1%–59.1%) alongside the confidence interval; (2) explicitly states that 'this comparison is limited by the absence of case-mix adjustment, the predominance of South African data, and the retrospective nature of all included studies'; (3) uses appropriately cautious language ('represent potential strategies' rather than 'are evidence-based strategies'); (4) identifies specific future research priorities including prospective multi-centre studies, studies from low-income countries, and cost-effectiveness analyses. See revised Conclusions section.

# RESPONSE TO REVIEWER #2

We are grateful to Reviewer #2 for their thorough and insightful critique. Their recommendation for major revision with substantial reframing has been carefully addressed, and we believe the manuscript is considerably strengthened as a result.

## Major Comments

**Major Comment 1:**

*"The fundamental limitation of proportional meta-analysis is that it pools crude mortality proportions without adjusting for case-mix (injury severity, patient demographics, indications for DCL). The authors should not attribute the mortality difference between LMICs and HICs primarily to 'health-system factors' without acknowledging that the patient populations may differ substantially. This requires major reframing of the Discussion and Conclusions."*

**Response:** We sincerely thank the reviewer for this critical observation, which we believe represents the most important improvement to our manuscript. We have undertaken a comprehensive reframing:

(1) Statistical Analysis section: Added an explicit paragraph stating that 'proportional meta-analysis pools crude mortality proportions and does not adjust for differences in case-mix, injury severity, or patient demographics across studies; this is an inherent limitation of the approach and the pooled estimate should be interpreted accordingly.'

(2) Discussion: Summary of Main Findings: Added two new paragraphs. The first addresses the case-mix limitation directly: 'Without individual patient data or standardised severity scoring, it is not possible to determine whether the higher pooled mortality reflects true health-system performance differences or differences in the populations undergoing DCL.' The second addresses the South African predominance.

(3) Discussion: Comparison with HIC Data: Changed 'The 2.6-fold mortality difference' to 'The observed difference in mortality' and added 'direct comparison is limited by several factors, including differences in study design, patient selection, and case-mix.'

(4) Discussion: Clinical Implications: Reframed from 'The finding that the LMIC-HIC mortality gap is driven primarily by modifiable health-system factors' to 'The observation that the LMIC-HIC mortality difference may be driven in part by health-system factors' with the addition of 'If confirmed by future studies with appropriate case-mix adjustment.'

(5) Limitations: Added a dedicated limitation (fifth) specifically addressing the absence of case-mix adjustment.

(6) Conclusions: Completely rewritten to explicitly state 'this comparison is limited by the absence of case-mix adjustment.'

See revised Methods: Statistical Analysis, Discussion (Summary, Comparison, Clinical Implications, Limitations), and Conclusions sections.

**Major Comment 2:**

*"The South African predominance (6/10 studies, 77% of patients) is a major issue that is not adequately discussed. South Africa, as an upper-middle-income country with established academic trauma centres, is not representative of LMICs broadly. The overall pooled estimate is essentially a South African estimate. This needs to be prominently acknowledged and discussed."*

**Response:** We fully agree and have made the following changes:

(1) Abstract: Added 'Six of the ten studies (n = 704, 77.0%) were from South Africa' to the Results section of the abstract.

(2) Results: Study Characteristics: Added a new prominent paragraph: 'Notably, South Africa contributed six of the ten studies and 77.0% (704/914) of the total patient population. This predominance should be considered when interpreting the pooled estimates, as the overall results are heavily weighted towards the South African experience.'

(3) Discussion: Summary of Main Findings: Added a new paragraph: 'the available evidence is heavily weighted towards South Africa... South African trauma centres represent a relatively well-resourced subset of LMIC settings, with established academic programmes and dedicated trauma units. The pooled estimate may therefore not be representative of DCL outcomes across the full spectrum of LMIC contexts.'

(4) Discussion: Interpretation of Heterogeneity: Added caveat that the zero heterogeneity within the South African subgroup 'may also reflect the relative homogeneity of the South African healthcare context... rather than a universal LMIC pattern.'

(5) Limitations: Expanded the fourth limitation to specifically state: 'South Africa, as an upper-middle-income country with established academic trauma centres, may not be representative of DCL outcomes in lower-middle-income or low-income settings.'

(6) Conclusions: Added 'the predominance of South African data in the available literature' as an explicit limitation of comparison.

See revised Abstract, Results, Discussion, Limitations, and Conclusions sections.

**Major Comment 3:**

*"The heterogeneity is very high (I² = 74.46%) and the prediction interval should be reported alongside the confidence interval to give a more realistic picture of the range of expected mortality rates. The current presentation of only the 95% CI gives a misleadingly narrow impression."*

**Response:** We agree completely. We have:

(1) Calculated and added the 95% prediction interval (20.1%–59.1%) throughout the manuscript, including the Abstract, Results: Primary Outcome, Discussion: Summary of Main Findings, and Conclusions.

(2) Added interpretation of the prediction interval: 'The 95% prediction interval indicates that a future comparable study from an LMIC setting could plausibly observe a mortality rate anywhere between approximately 20% and 59%.'

(3) Added the prediction interval as a measure of heterogeneity in the Statistical Analysis section.

(4) Added a new paragraph in the Statistical Analysis section explaining the rationale for reporting prediction intervals.

The prediction interval clearly demonstrates the wide range of plausible outcomes across LMIC settings, providing important context that was missing from the original manuscript. See revised Abstract, Methods: Statistical Analysis, Results: Primary Outcome, Discussion, and Conclusions sections.

**Major Comment 4:**

*"The conclusions are too strong given the evidence base. Phrases like 'The mortality gap is driven primarily by health-system factors' and 'evidence-based strategies to close the LMIC-HIC outcome gap' are not supported by the data from a proportional meta-analysis of crude rates. These should be substantially toned down."*

**Response:** We have substantially revised all overly strong claims:

(1) Removed 'The mortality gap is driven primarily by health-system factors' and replaced with 'The observation that the LMIC-HIC mortality difference may be driven in part by health-system factors.'

(2) Changed 'evidence-based strategies to close the LMIC-HIC outcome gap' to 'potential strategies to improve outcomes, though the evidence base for these recommendations remains indirect.'

(3) Changed 'approximately 2.6 times higher than in high-income countries' to 'numerically higher than reported HIC benchmarks. However, this comparison is limited by the absence of case-mix adjustment.'

(4) Changed 'are remarkably consistent' to 'may be relatively consistent.'

(5) Changed 'constrains' to 'may constrain' and 'limit' to 'may limit' in the Discussion.

(6) Added 'These recommendations are offered tentatively and will require validation in prospective studies.'

(7) Added 'If confirmed by future studies with appropriate case-mix adjustment' before listing interventions.

See revised Discussion and Conclusions sections throughout.

## Minor Comments

**Minor Comment 1:**

*"The data availability statement should specify that all data are within the article and supplementary material, rather than 'available from the corresponding author on reasonable request', which is not appropriate for a systematic review of published data."*

**Response:** Corrected. The Data Availability Statement now reads: 'All data underlying the findings of this systematic review are available within the article and its Supplementary Material. The extracted data from included studies are presented in Table 1. The complete search strategy is provided in Supplementary Material S1.' See revised Data Availability Statement.

**Minor Comment 2:**

*"Tables should be incorporated into the main manuscript rather than submitted as separate files."*

**Response:** Done. Table 1 (Characteristics of included studies) and Table 2 (Risk of bias assessment) have been incorporated into the main manuscript within the Results: Study Characteristics section. The separate table files have been removed.

**Minor Comment 3:**

*"The ethics statement appears both in the Methods and in the end-matter declarations. It should appear in the Methods section only, per PLOS ONE requirements."*

**Response:** Corrected. The ethics statement now appears only in the Methods section (as a new subsection after the protocol registration statement) and has been removed from the end-matter declarations. See revised Methods: Ethics Statement section.

**Minor Comment 4:**

*"The supplementary material should have proper captions at the end of the manuscript."*

**Response:** Added. Proper captions for all supplementary material have been added at the end of the manuscript, including: 'Supplementary Material S1. Detailed search strategy for each database' and 'Table S1. Complete search strategy for PubMed/MEDLINE, Scopus, Google Scholar, Cochrane Library, and African Journals Online (AJOL), including search terms, Boolean operators, filters, and number of records retrieved per database.' See end of revised manuscript.

**Minor Comment 5:**

*"Please provide the detailed search strategy, either in the manuscript or as supplementary material."*

**Response:** The detailed search strategy has been prepared as Supplementary Material S1 (Table S1), containing the complete search strings for each of the five databases. A reference to this supplementary table has been added in the Methods: Search Strategy section: 'The complete search strategy for each database is provided in Supplementary Material S1 (Table S1).' See revised Methods: Search Strategy section and Supplementary Material S1.

**Minor Comment 6:**

*"Some language in the Discussion could be more cautious when comparing LMIC and HIC outcomes. Several assertions about health-system factors being the 'primary' driver of the mortality gap are not directly supported by the data."*

**Response:** We have carefully reviewed all language in the Discussion and have: (1) replaced 'is driven primarily by' with 'may be driven in part by'; (2) added hedging phrases such as 'may plausibly contribute', 'potentially attributable to', and 'if confirmed by future studies'; (3) added caveats about case-mix differences wherever comparisons are made; (4) qualified clinical recommendations as 'tentative' pending further evidence. See revised Discussion sections throughout.

We hope that these revisions adequately address the concerns raised by the Editor and both Reviewers. We believe the manuscript has been substantially strengthened by this process, particularly through the more cautious interpretation of findings, the explicit acknowledgement of the case-mix limitation, the prominent discussion of South African predominance, and the addition of prediction intervals. We are grateful for the opportunity to revise and resubmit this work.

Sincerely,

Shumani Makhadi

Corresponding author

On behalf of all authors
