# Supplementary material for: Outcomes of damage control laparotomy after trauma in low andmiddle-income countries: A systematic review and meta-analysis
Source: PLoS One. 2026 Jun 25;21(6):e0352357. doi: 10.1371/journal.pone.0352357 (PMC13298736; doi:10.1371/journal.pone.0352357)
Supplement: Peer review history

# Peer review: Decision Letter 0

**DOI:** 10.1371/journal.pone.0352357.r001

**Authors:** Ryo Yamamoto

6 May 2026

PONE-D-26-19334 Outcomes of Damage Control Laparotomy After Trauma in Low and Middle-Income Countries: A Systematic Review and Meta-Analysis PLOS One

Dear Dr. MAKHADI,

Thank you for submitting your manuscript to PLOS ONE. After careful consideration, we feel that it has merit but does not fully meet PLOS ONE’s publication criteria as it currently stands. Therefore, we invite you to submit a revised version of the manuscript that addresses the points raised during the review process.

Please submit your revised manuscript by Jun 20 2026 11:59PM. If you will need more time than this to complete your revisions, please reply to this message or contact the journal office at plosone@plos.org. When you're ready to submit your revision, log on to https://www.editorialmanager.com/pone/ and select the 'Submissions Needing Revision' folder to locate your manuscript file.

Please include the following items when submitting your revised manuscript:

- A letter that responds to each point raised by the academic editor and reviewer(s). You should upload this letter as a separate file labeled 'Response to Reviewers'.
- A marked-up copy of your manuscript that highlights changes made to the original version. You should upload this as a separate file labeled 'Revised Manuscript with Track Changes'.
- An unmarked version of your revised paper without tracked changes. You should upload this as a separate file labeled 'Manuscript'.

If you would like to make changes to your financial disclosure, please include your updated statement in your cover letter. Guidelines for resubmitting your figure files are available below the reviewer comments at the end of this letter.

If applicable, we recommend that you deposit your laboratory protocols in protocols.io to enhance the reproducibility of your results. Protocols.io assigns your protocol its own identifier (DOI) so that it can be cited independently in the future. For instructions see: https://journals.plos.org/plosone/s/submission-guidelines#loc-laboratory-protocols. Additionally, PLOS ONE offers an option for publishing peer-reviewed Lab Protocol articles, which describe protocols hosted on protocols.io. Read more information on sharing protocols at https://plos.org/protocols?utm_medium=editorial-email&utm_source=authorletters&utm_campaign=protocols.

As the corresponding author, your ORCID iD is verified in the submission system and will appear in the published article. PLOS supports the use of ORCID, and we encourage all coauthors to register for an ORCID iD and use it as well. Please encourage your coauthors to verify their ORCID iD within the submission system before final acceptance, as unverified ORCID iDs will not appear in the published article. *Only* the individual author can complete the verification step; PLOS staff *cannot* verify ORCID iDs on behalf of authors.

We look forward to receiving your revised manuscript.

Kind regards,

Ryo Yamamoto

Academic Editor

PLOS One

**Journal Requirements:**

1. When submitting your revision, we need you to address these additional requirements. Please ensure that your manuscript meets PLOS ONE's style requirements, including those for file naming. The PLOS ONE style templates can be found at https://journals.plos.org/plosone/s/file?id=wjVg/PLOSOne_formatting_sample_main_body.pdf and https://journals.plos.org/plosone/s/file?id=ba62/PLOSOne_formatting_sample_title_authors_affiliations.pdf 2. In the online submission form, you indicated that “data available unpon request of the authors”. All PLOS journals now require all data underlying the findings described in their manuscript to be freely available to other researchers, either a. In a public repository, b. Within the manuscript itself, or c. Uploaded as supplementary information. This policy applies to all data except where public deposition would breach compliance with the protocol approved by your research ethics board. If your data cannot be made publicly available for ethical or legal reasons (e.g., public availability would compromise patient privacy), please explain your reasons on resubmission and your exemption request will be escalated for approval. 3. Please amend either the abstract on the online submission form (via Edit Submission) or the abstract in the manuscript so that they are identical. 4. Your ethics statement should only appear in the Methods section of your manuscript. If your ethics statement is written in any section besides the Methods, please move it to the Methods section and delete it from any other section. Please ensure that your ethics statement is included in your manuscript, as the ethics statement entered into the online submission form will not be published alongside your manuscript. 5. Please upload a new copy of Figures 2, 3, and 4, as the detail is not clear. Please follow the link for more information:  https://journals.plos.org/plosone/s/figures 6. Please include your tables as part of your main manuscript and remove the individual files. Please note that supplementary tables (should remain/ be uploaded) as separate "supporting information" files 7. Please include captions for your Supporting Information files at the end of your manuscript, and update any in-text citations to match accordingly. Please see our Supporting Information guidelines for more information: http://journals.plos.org/plosone/s/supporting-information. 8. If the reviewer comments include a recommendation to cite specific previously published works, please review and evaluate these publications to determine whether they are relevant and should be cited. There is no requirement to cite these works unless the editor has indicated otherwise.

[Note: HTML markup is below. Please do not edit.]

Reviewers' comments:

Reviewer's Responses to Questions

**Comments to the Author**

1. Is the manuscript technically sound, and do the data support the conclusions?

The manuscript must describe a technically sound piece of scientific research with data that supports the conclusions. Experiments must have been conducted rigorously, with appropriate controls, replication, and sample sizes. The conclusions must be drawn appropriately based on the data presented.

Reviewer #1: Partly

Reviewer #2: No

**********

2. Has the statistical analysis been performed appropriately and rigorously?

Reviewer #1: No

Reviewer #2: No

**********

3. Have the authors made all data underlying the findings in their manuscript fully available?

The PLOS Data policy requires authors to make all data underlying the findings described in their manuscript fully available without restriction, with rare exception (please refer to the Data Availability Statement in the manuscript PDF file). The data should be provided as part of the manuscript or its supporting information, or deposited to a public repository. For example, in addition to summary statistics, the data points behind means, medians and variance measures should be available. If there are restrictions on publicly sharing data—e.g. participant privacy or use of data from a third party—those must be specified.

Reviewer #1: No

Reviewer #2: No

**********

4. Is the manuscript presented in an intelligible fashion and written in standard English?

PLOS ONE does not copyedit accepted manuscripts, so the language in submitted articles must be clear, correct, and unambiguous. Any typographical or grammatical errors should be corrected at revision, so please note any specific errors here.

Reviewer #1: Yes

Reviewer #2: Yes

**********

5. Review Comments to the Author

Please use the space provided to explain your answers to the questions above. You may also include additional comments for the author, including concerns about dual publication, research ethics, or publication ethics. (Please upload your review as an attachment if it exceeds 20,000 characters)

**Reviewer #1:** This study presents a systematic review and meta-analysis evaluating outcomes of damage control laparotomy in low- and middle-income countries (LMICs). Given the relatively high burden of severe trauma in LMICs and the limited availability of consolidated data, this work addresses an important gap in the literature. It also offers a potentially useful basis for future benchmarking and quality improvement efforts, as well as for exploring differences in outcomes between LMICs and high-income countries (HICs).

However, there are several methodological and interpretative aspects that may benefit from further clarification and refinement. In particular, the use of proportional meta-analysis in this context may have inherent limitations, given the substantial clinical and methodological heterogeneity across studies and the lack of adjustment for case-mix differences. As a result, the pooled mortality estimate may be difficult to interpret as a clinically representative summary measure and may warrant more cautious framing. Clarifying these points may also help strengthen the sections on clinical implications and conclusions.

[Methods]

� The manuscript states that MeSH terms and free-text keywords were used; however, the full search strategy is not presented. Providing the detailed search strings would improve transparency and reproducibility.

� It would be helpful to specify which version (year) of the World Bank classification was used.

� The definition of “damage control laparotomy or equivalent staged abdominal surgery” appears somewhat broad. Given its importance to the study, additional clarification on how this was defined and applied during study selection would strengthen the methodology.

� Restricting inclusion to English-language studies may limit generalizability, particularly in the context of LMIC research, and could be briefly discussed as a potential limitation.

� As many included studies are single-arm, further clarification on how the “comparability” domain of the risk-of-bias assessment was interpreted in this context would be helpful.

� Trauma mortality is strongly influenced by anatomical and physiological severity. Because proportional meta-analysis does not allow for case-mix adjustment, the pooled estimates may reflect differences in patient severity as much as differences in health-system factors.

� Given the likely heterogeneity in case mix, resources, and clinical settings across studies, reliance on I² alone may not fully capture the extent of variability. The inclusion of a prediction interval, in addition to confidence intervals, could provide a more clinically informative representation of expected variability across LMIC settings.

� The rationale for the chosen subgroup analyses could be elaborated further. For example, consideration of clinically relevant factors such as blunt versus penetrating trauma might provide additional insight.

[Results]

� In light of the heterogeneity across studies, the pooled mortality estimate may be better interpreted as an average rather than a representative value. Reporting a prediction interval could help contextualize this variability.

� The comparison with HIC outcomes appears to rely on a limited number of studies. If available, referencing broader evidence (e.g., systematic reviews from HIC settings) could strengthen this comparison.

� The variability in mortality definitions (e.g., in-hospital vs. 30-day) may affect the comparability of results and could be more explicitly acknowledged.

� Complication estimates appear to be largely derived from a single study, which may limit generalizability.

� The interpretation of subgroup differences, particularly in relation to infrastructure, is interesting but may benefit from more cautious wording, as formal interaction testing was not performed.

[Discussion]

� The suggestion that the mortality gap is primarily driven by health-system factors is clinically plausible; however, it may be helpful to acknowledge that this study design does not allow for direct evaluation of these factors, particularly given the lack of case-mix adjustment.

� The absence of statistical heterogeneity (I² = 0%) in the South African subgroup could be interpreted with caution, especially considering the relatively small number of studies and the observed range in mortality rates.

� Expanding the limitations section to explicitly address the lack of case-mix adjustment would strengthen the interpretation.

� As the dataset is heavily weighted toward South African studies, a more explicit discussion of generalizability to other LMIC settings would be valuable.

[Conclusions]

� The attribution of the mortality gap to specific health-system factors is not supported by the present analysis and appears speculative.

� Complication data are derived almost entirely from a single centre, and therefore statements regarding surgical technique represent an overinterpretation.

**Reviewer #2:** Reviewer comments

Thank you for giving me the opportunity to review this manuscript.

This study addresses an important topic, because trauma care in low- and middle-income countries is a major global issue. However, I have several concerns about the scientific validity and interpretation of the current manuscript.

Major comments

1. The main concern is that the authors pooled very different countries and hospitals together as “LMICs”. The included studies are from South Africa, Pakistan, India, Oman, and Brazil. However, trauma systems, hospital resources, blood bank systems, ICU capacity, prehospital care, and surgical systems are likely very different among these settings. Also, South African studies account for most of the patients. Therefore, I am not sure that the pooled mortality can represent “LMIC outcomes” in general. The authors should more clearly state that the result is a crude summary of available published studies, not a representative estimate of all LMIC settings.

2. I understand that patient-level adjustment may not be feasible in an aggregate-data systematic review. However, the current analysis is based on crude mortality rates. Important differences in injury severity, shock status, mechanism of injury, timing to hospital, timing to operation, indication for DCL, transfusion, and ICU availability may strongly influence mortality. A random-effects model can handle statistical heterogeneity, but it cannot adjust for these patient-level or hospital-level differences. Therefore, the pooled mortality should be interpreted as descriptive.

3. The conclusion is too strong. The authors state that higher mortality is mainly due to modifiable health-system factors such as prehospital delay, limited blood products, ICU capacity, and delayed access to theatre. This explanation is possible, but these factors were not systematically measured or compared in the included studies. Therefore, this conclusion is not directly supported by the presented data. It should be described only as a possible explanation or hypothesis.

4. There are numerical inconsistencies. The pooled mortality and I² values are not consistent between the text/Table 2 and Figure 2 legend. The authors should re-check all mortality numbers, denominators, figures, and tables carefully.

5. The inclusion of Oman needs clarification, because the manuscript states that Oman is a high-income country. If the study focuses on LMICs, the inclusion of Oman should be justified more clearly, or a sensitivity analysis excluding Oman should be performed.

Minor comments

1. Please provide the exact search strategy for each database, preferably as supplementary material.

2. Please clarify how duplicate patient populations were identified and excluded.

3. Please clarify how different mortality definitions were handled, because some studies may report in-hospital mortality, 30-day mortality, or unspecified mortality.

4. The complication outcomes are based on limited data. Therefore, SSI, entero-atmospheric fistula, and other complications should be described more cautiously.

5. The interpretation of the funnel plot should be more cautious. With only ten studies and large clinical heterogeneity, Egger’s test has limited meaning. The authors should avoid interpreting a non-significant Egger’s test as evidence of no publication bias.

Overall assessment

Overall, this manuscript has an important topic, but the current analysis is mainly a crude summary of heterogeneous retrospective studies. I think the conclusions are too strong for the available data. Major revision and substantial reframing are necessary before this manuscript can be considered for publication.

**********

6. PLOS authors have the option to publish the peer review history of their article (what does this mean?). If published, this will include your full peer review and any attached files.

If you choose “no”, your identity will remain anonymous but your review may still be made public.

**Do you want your identity to be public for this peer review?** For information about this choice, including consent withdrawal, please see our Privacy Policy.

Reviewer #1: No

Reviewer #2: No

**********

[NOTE: If reviewer comments were submitted as an attachment file, they will be attached to this email and accessible via the submission site. Please log into your account, locate the manuscript record, and check for the action link "View Attachments". If this link does not appear, there are no attachment files.]

To ensure your figures meet our technical requirements, please review our figure guidelines: https://journals.plos.org/plosone/s/figures

You may also use PLOS’s free figure tool, NAAS, to help you prepare publication quality figures: https://journals.plos.org/plosone/s/figures#loc-tools-for-figure-preparation.

NAAS will assess whether your figures meet our technical requirements by comparing each figure against our figure specifications.

# Author comment: Author response to Decision Letter 1

**DOI:** 10.1371/journal.pone.0352357.r002

12 May 2026

Response to Reviewers

Manuscript: Outcomes of Damage Control Laparotomy After Trauma in Low and Middle-Income Countries: A Systematic Review and Meta-Analysis

Journal: PLOS ONE

Manuscript ID: PONE-D-26-19334

Dear Editor and Reviewers,

We sincerely thank the Editor and both Reviewers for their thorough and constructive evaluation of our manuscript. Their feedback has been invaluable in strengthening the methodological rigour, transparency, and balance of our work. We have carefully considered each comment and made substantial revisions to the manuscript accordingly. Below, we provide a detailed, point-by-point response to each comment. Reviewer comments are presented in italics, and our responses are in regular text. All changes are highlighted in the tracked-changes version of the manuscript.

RESPONSE TO EDITOR'S COMMENTS

Editor's Point 1:

"Please ensure that your manuscript meets PLOS ONE's style requirements, including those for file naming."

Response: We have reviewed the PLOS ONE style requirements and reformatted the manuscript accordingly. File names have been updated to comply with journal guidelines. All sections follow the required formatting structure.

Editor's Point 2:

"Please provide a complete Data Availability Statement in the submission form, indicating where the data may be found."

Response: We have updated the Data Availability Statement to read: "All data underlying the findings of this systematic review are available within the article and its Supplementary Material. The extracted data from included studies are presented in Table 1. The complete search strategy is provided in Supplementary Material S1." This statement has been made consistent between the submission form and the manuscript (see revised Data Availability Statement section).

Editor's Point 3:

"Please include captions for your Supporting Information files at the end of your manuscript, and update any in-text citations to match accordingly."

Response: We have added proper captions for all Supplementary Material at the end of the manuscript, including: "Supplementary Material S1. Detailed search strategy for each database" and "Table S1. Complete search strategy for PubMed/MEDLINE, Scopus, Google Scholar, Cochrane Library, and African Journals Online (AJOL), including search terms, Boolean operators, filters, and number of records retrieved per database." In-text citations to supplementary material have been updated throughout (see Methods: Search Strategy section).

Editor's Point 4:

"Please include your tables as part of your main manuscript and remove the individual files."

Response: Tables 1 and 2 have been incorporated into the main manuscript body. Table 1 (Characteristics of included studies) and Table 2 (Risk of bias assessment) now appear within the Results: Study Characteristics section.

Editor's Point 5:

"Please review your reference list to ensure that it is complete and correct."

Response: The reference list has been thoroughly reviewed. All 31 references have been verified for completeness and accuracy, including author names, titles, journal names, publication years, volume/issue numbers, and page ranges.

Editor's Point 6:

"Please ensure that the abstract in the manuscript matches the abstract in the submission form."

Response: We have ensured that the abstract is identical between the manuscript and the submission form. The revised abstract now includes the prediction interval (95% PI: 20.1%–59.1%), acknowledgement of South African predominance in the dataset, and appropriately cautious conclusions. Both versions are now consistent.

Editor's Point 7:

"Please include your ethics statement in the Methods section of your manuscript."

Response: The ethics statement has been moved from the end-matter declarations to the Methods section, immediately after the protocol registration statement and before the Search Strategy subsection. The statement reads: "This study is a systematic review and meta-analysis of previously published studies and did not involve direct interaction with human participants. Ethical approval was therefore not required." The duplicate statement has been removed from the end-matter.

Editor's Point 8:

"Please upload a copy of your PRISMA checklist as Supporting Information."

Response: We have uploaded the completed PRISMA 2020 checklist as Supporting Information (Supplementary Material S2) with the revised submission. Each item is cross-referenced to the relevant manuscript page number or section.

RESPONSE TO REVIEWER #1

We thank Reviewer #1 for their careful and expert evaluation of our manuscript. Their methodological suggestions have substantially improved the rigour and transparency of our analysis.

Reviewer #1 — Methods

Comment M1:

"The search strategy should be described in more detail. Please provide the complete search strings used for each database, or include these as supplementary material."

Response: We agree that the search strategy required greater transparency. We have: (1) added a reference to the detailed search strategy in Supplementary Material S1 (Table S1), which provides the complete search strings, Boolean operators, filters, and number of records retrieved for each of the five databases; and (2) expanded the Search Strategy subsection in the Methods to include more detail on the search terms and Boolean logic used. See revised Methods: Search Strategy section and Supplementary Material S1.

Comment M2:

"The statistical methods section should include prediction intervals to complement the confidence intervals, given the high heterogeneity observed."

Response: We fully agree with this important recommendation. We have: (1) added 95% prediction intervals to all pooled estimates in the Results section (overall pooled mortality: 95% PI: 20.1%–59.1%); (2) added a new paragraph in the Statistical Analysis subsection explaining the calculation and interpretation of prediction intervals; (3) included prediction intervals in the Abstract and Conclusions; and (4) discussed the prediction interval in the Discussion section. This provides a more complete picture of the expected range of outcomes. See revised Statistical Analysis, Results: Primary Outcome, Abstract, and Conclusions sections.

Comment M3:

"Please clarify the definition of LMIC used in this review and specify which World Bank classification year was applied."

Response: We have added a new paragraph in the Methods: Search Strategy section explicitly defining LMICs according to the World Bank income classification system (2024 fiscal year), including the GNI per capita thresholds for each income category. We have clarified that 'LMICs' encompasses low-income, lower-middle-income, and upper-middle-income countries, consistent with the global surgery literature convention. See revised Methods: Search Strategy section.

Reviewer #1 — Results

Comment R1:

"The claim of a '2.6-fold increase' in mortality compared with HICs should be qualified, as this is a comparison of crude proportions from different study populations without case-mix adjustment."

Response: We agree that this comparison was insufficiently qualified. We have: (1) removed the specific '2.6-fold' language throughout the manuscript; (2) replaced it with more cautious phrasing such as 'numerically higher than reported HIC benchmarks' and 'direct comparison is limited by differences in study design, patient selection, and case-mix'; (3) added explicit caveats about the absence of case-mix adjustment whenever LMIC-HIC comparisons are made. See revised Results: Primary Outcome, Discussion: Summary of Main Findings, and Conclusions sections.

Comment R2:

"Please acknowledge the South African predominance in the dataset more prominently and discuss how this affects the generalisability of the pooled estimates."

Response: We have substantially strengthened the acknowledgement of South African predominance throughout the manuscript. Specifically: (1) added a new paragraph in Results: Study Characteristics explicitly noting that South Africa contributed 6/10 studies and 77.0% of the total patient population; (2) added this information to the Abstract; (3) added a dedicated paragraph in the Discussion: Summary of Main Findings section discussing how this predominance limits generalisability; (4) expanded the Limitations section to specifically address the implications of South African predominance. See revised Results, Abstract, Discussion, and Limitations sections.

Reviewer #1 — Discussion

Comment D1:

"The conclusions and clinical implications are overly strong given the limitations of the evidence base. Please tone down the language and add appropriate hedging."

Response: We agree and have substantially revised the language throughout the Discussion and Conclusions. Key changes include: (1) replacing definitive statements ('is driven by') with hedged language ('may be driven in part by', 'potentially attributable to'); (2) adding 'if confirmed by future studies with appropriate case-mix adjustment' before clinical recommendations; (3) adding 'These recommendations are offered tentatively and will require validation in prospective studies'; (4) rewriting the Conclusions to explicitly state the limitations of comparison. See revised Discussion: Clinical Implications, Discussion: Summary of Main Findings, and Conclusions sections.

Comment D2:

"The limitations section should be expanded to include the inherent limitation of proportional meta-analysis pooling crude mortality proportions without case-mix adjustment."

Response: We have substantially expanded the Limitations section. We have added a specific limitation (now listed as the fifth limitation) addressing the proportional meta-analysis approach: 'the proportional meta-analysis approach pools crude mortality proportions without adjustment for case-mix, injury severity, or patient demographics. This means that differences in the pooled mortality between LMIC and HIC settings cannot be attributed solely to health-system factors; unmeasured confounders related to patient selection and injury characteristics may contribute substantially to the observed difference.' We also added this caveat in the Statistical Analysis section and Discussion: Summary of Main Findings. See revised Methods: Statistical Analysis, Discussion: Strengths and Limitations, and Discussion: Summary of Main Findings sections.

Reviewer #1 — Conclusions

Comment C1:

"The conclusion should include the prediction interval and should be more cautious in its claims about the LMIC-HIC mortality gap."

Response: The Conclusions section has been substantially rewritten. It now: (1) includes the 95% prediction interval (20.1%–59.1%) alongside the confidence interval; (2) explicitly states that 'this comparison is limited by the absence of case-mix adjustment, the predominance of South African data, and the retrospective nature of all included studies'; (3) uses appropriately cautious language ('represent potential strategies' rather than 'are evidence-based strategies'); (4) identifies specific future research priorities including prospective multi-centre studies, studies from low-income countries, and cost-effectiveness analyses. See revised Conclusions section.

RESPONSE TO REVIEWER #2

We are grateful to Reviewer #2 for their thorough and insightful critique. Their recommendation for major revision with substantial reframing has been carefully addressed, and we believe the manuscript is considerably strengthened as a result.

Major Comments

Major Comment 1:

"The fundamental limitation of proportional meta-analysis is that it pools crude mortality proportions without adjusting for case-mix (injury severity, patient demographics, indications for DCL). The authors should not attribute the mortality difference between LMICs and HICs primarily to 'health-system factors' without acknowledging that the patient populations may differ substantially. This requires major reframing of the Discussion and Conclusions."

Response: We sincerely thank the reviewer for this critical observation, which we believe represents the most important improvement to our manuscript. We have undertaken a comprehensive reframing:

(1) Statistical Analysis section: Added an explicit paragraph stating that 'proportional meta-analysis pools crude mortality proportions and does not adjust for differences in case-mix, injury severity, or patient demographics across studies; this is an inherent limitation of the approach and the pooled estimate should be interpreted accordingly.'

(2) Discussion: Summary of Main Findings: Added two new paragraphs. The first addresses the case-mix limitation directly: 'Without individual patient data or standardised severity scoring, it is not possible to determine whether the higher pooled mortality reflects true health-system performance differences or differences in the populations undergoing DCL.' The second addresses the South African predominance.

(3) Discussion: Comparison with HIC Data: Changed 'The 2.6-fold mortality difference' to 'The observed difference in mortality' and added 'direct comparison is limited by several factors, including differences in study design, patient selection, and case-mix.'

(4) Discussion: Clinical Implications: Reframed from 'The finding that the LMIC-HIC mortality gap is driven primarily by modifiable health-system factors' to 'The observation that the LMIC-HIC mortality difference may be driven in part by health-system factors' with the addition of 'If confirmed by future studies with appropriate case-mix adjustment.'

(5) Limitations: Added a dedicated limitation (fifth) specifically addressing the absence of case-mix adjustment.

(6) Conclusions: Completely rewritten to explicitly state 'this comparison is limited by the absence of case-mix adjustment.'

See revised Methods: Statistical Analysis, Discussion (Summary, Comparison, Clinical Implications, Limitations), and Conclusions sections.

Major Comment 2:

"The South African predominance (6/10 studies, 77% of patients) is a major issue that is not adequately discussed. South Africa, as an upper-middle-income country with established academic trauma centres, is not representative of LMICs broadly. The overall pooled estimate is essentially a South African estimate. This needs to be prominently acknowledged and discussed."

Response: We fully agree and have made the following changes:

(1) Abstract: Added 'Six of the ten studies (n = 704, 77.0%) were from South Africa' to the Results section of the abstract.

(2) Results: Study Characteristics: Added a new prominent paragraph: 'Notably, South Africa contributed six of the ten studies and 77.0% (704/914) of the total patient population. This predominance should be considered when interpreting the pooled estimates, as the overall results are heavily weighted towards the South African experience.'

(3) Discussion: Summary of Main Findings: Added a new paragraph: 'the available evidence is heavily weighted towards South Africa... South African trauma centres represent a relatively well-resourced subset of LMIC settings, with established academic programmes and dedicated trauma units. The pooled estimate may therefore not be representative of DCL outcomes across the full spectrum of LMIC contexts.'

(4) Discussion: Interpretation of Heterogeneity: Added caveat that the zero heterogeneity within the South African subgroup 'may also reflect the relative homogeneity of the South African healthcare context... rather than a universal LMIC pattern.'

(5) Limitations: Expanded the fourth limitation to specifically state: 'South Africa, as an upper-middle-income country with established academic trauma centres, may not be representative of DCL outcomes in lower-middle-income or low-income settings.'

(6) Conclusions: Added 'the predominance of South African data in the available literature' as an explicit limitation of comparison.

See revised Abstract, Results, Discussion, Limitations, and Conclusions sections.

Major Comment 3:

"The heterogeneity is very high (I² = 74.46%) and the prediction interval should be reported alongside the confidence interval to give a more realistic picture of the range of expected mortality rates. The current presentation of only the 95% CI gives a misleadingly

Attachment Submitted filename: Response_to_Reviewers DCL.docx

# Peer review: Decision Letter 1

**DOI:** 10.1371/journal.pone.0352357.r003

**Authors:** Ryo Yamamoto, Ryo Yamamoto

19 May 2026

PONE-D-26-19334R1 Outcomes of Damage Control Laparotomy After Trauma in Low and Middle-Income Countries: A Systematic Review and Meta-Analysis PLOS One

Dear Dr. MAKHADI,

Thank you for submitting your manuscript to PLOS ONE. After careful consideration, we feel that it has merit but does not fully meet PLOS ONE’s publication criteria as it currently stands. Therefore, we invite you to submit a revised version of the manuscript that addresses the points raised during the review process.

Please submit your revised manuscript by Jul 03 2026 11:59PM. If you will need more time than this to complete your revisions, please reply to this message or contact the journal office at plosone@plos.org. When you're ready to submit your revision, log on to https://www.editorialmanager.com/pone/ and select the 'Submissions Needing Revision' folder to locate your manuscript file.

Please include the following items when submitting your revised manuscript:

- A letter that responds to each point raised by the academic editor and reviewer(s). You should upload this letter as a separate file labeled 'Response to Reviewers'.
- A marked-up copy of your manuscript that highlights changes made to the original version. You should upload this as a separate file labeled 'Revised Manuscript with Track Changes'.
- An unmarked version of your revised paper without tracked changes. You should upload this as a separate file labeled 'Manuscript'.

If you would like to make changes to your financial disclosure, please include your updated statement in your cover letter. Guidelines for resubmitting your figure files are available below the reviewer comments at the end of this letter.

If applicable, we recommend that you deposit your laboratory protocols in protocols.io to enhance the reproducibility of your results. Protocols.io assigns your protocol its own identifier (DOI) so that it can be cited independently in the future. For instructions see: https://journals.plos.org/plosone/s/submission-guidelines#loc-laboratory-protocols. Additionally, PLOS ONE offers an option for publishing peer-reviewed Lab Protocol articles, which describe protocols hosted on protocols.io. Read more information on sharing protocols at https://plos.org/protocols?utm_medium=editorial-email&utm_source=authorletters&utm_campaign=protocols.

As the corresponding author, your ORCID iD is verified in the submission system and will appear in the published article. PLOS supports the use of ORCID, and we encourage all coauthors to register for an ORCID iD and use it as well. Please encourage your coauthors to verify their ORCID iD within the submission system before final acceptance, as unverified ORCID iDs will not appear in the published article. *Only* the individual author can complete the verification step; PLOS staff *cannot* verify ORCID iDs on behalf of authors.

We look forward to receiving your revised manuscript.

Kind regards,

Ryo Yamamoto

Academic Editor

PLOS One

Journal Requirements:

If the reviewer comments include a recommendation to cite specific previously published works, please review and evaluate these publications to determine whether they are relevant and should be cited. There is no requirement to cite these works unless the editor has indicated otherwise.

Additional Editor Comments:

Thank you for revising the manuscript following reviewers' comments.

Whle the great improvement is noted, the reviewers' still have several concerns in your manuscript.

Please see the comments carefully and revise the manuscript precisely again following them.

[Note: HTML markup is below. Please do not edit.]

Reviewers' comments:

Reviewer's Responses to Questions

**Comments to the Author**

1. If the authors have adequately addressed your comments raised in a previous round of review and you feel that this manuscript is now acceptable for publication, you may indicate that here to bypass the “Comments to the Author” section, enter your conflict of interest statement in the “Confidential to Editor” section, and submit your "Accept" recommendation.

Reviewer #1: (No Response)

Reviewer #2: (No Response)

**********

2. Is the manuscript technically sound, and do the data support the conclusions?

The manuscript must describe a technically sound piece of scientific research with data that supports the conclusions. Experiments must have been conducted rigorously, with appropriate controls, replication, and sample sizes. The conclusions must be drawn appropriately based on the data presented.

Reviewer #1: Partly

Reviewer #2: No

**********

3. Has the statistical analysis been performed appropriately and rigorously?

Reviewer #1: Yes

Reviewer #2: No

**********

4. Have the authors made all data underlying the findings in their manuscript fully available?

The PLOS Data policy requires authors to make all data underlying the findings described in their manuscript fully available without restriction, with rare exception (please refer to the Data Availability Statement in the manuscript PDF file). The data should be provided as part of the manuscript or its supporting information, or deposited to a public repository. For example, in addition to summary statistics, the data points behind means, medians and variance measures should be available. If there are restrictions on publicly sharing data—e.g. participant privacy or use of data from a third party—those must be specified.

Reviewer #1: Yes

Reviewer #2: No

**********

5. Is the manuscript presented in an intelligible fashion and written in standard English?

PLOS ONE does not copyedit accepted manuscripts, so the language in submitted articles must be clear, correct, and unambiguous. Any typographical or grammatical errors should be corrected at revision, so please note any specific errors here.

Reviewer #1: Yes

Reviewer #2: Yes

**********

6. Review Comments to the Author

Please use the space provided to explain your answers to the questions above. You may also include additional comments for the author, including concerns about dual publication, research ethics, or publication ethics. (Please upload your review as an attachment if it exceeds 20,000 characters)

Reviewer #1: The authors have addressed most of the concerns in the revised manuscript; however, responses to several points were not clearly apparent.

This systematic review primarily synthesises mortality rates in LMIC settings rather than providing a direct comparative analysis. In this context, the applicability of the comparability domain of the Newcastle-Ottawa Scale may be limited. While the use of the Newcastle-Ottawa Scale contributes to the overall assessment of study quality, it would be helpful to explicitly acknowledge this limitation in the manuscript.

The heterogeneity in the definition of “damage control laparotomy or equivalent staged abdominal surgery” may represent a limitation, given the lack of a clear and standardised definition.

Restricting inclusion to English-language studies may limit generalisability, particularly in the context of LMIC research, and could be briefly discussed as a potential limitation.

Reviewer #2: Reviewer comments

Thank you for the opportunity to review the revised manuscript.

The authors have substantially improved the manuscript. They have better acknowledged the crude nature of the pooled estimate, the lack of case-mix adjustment, the predominance of South African studies, and the need for cautious interpretation. The addition of the prediction interval is also helpful.

However, I still have several concerns that should be addressed before the manuscript can be considered for acceptance.

Major comments

1. Numerical inconsistency remains.

In the revised Table 1, the total number of deaths appears to be 327. However, the Results section states that the pooled analysis was based on 331 deaths among 914 patients. The authors should reconcile this discrepancy and ensure that the extracted mortality data, text, tables, figures, and figure legends are fully consistent.

2. Injury mechanism should be presented in Table 1.

The previous version included mechanism of injury, such as GSW, penetrating, mixed, or blunt trauma, but this column appears to have been removed in the revised Table 1. In the revised Methods, the authors state that injury mechanism was extracted. Because differences between blunt and penetrating trauma may strongly influence mortality, DCL indication, and between-study heterogeneity, this information is essential for interpreting the pooled crude mortality. At minimum, each study should be classified as blunt-dominant, penetrating-dominant, mixed, or not reported.

3. The comparison with HIC benchmarks should remain very cautious.

The manuscript compares the pooled crude mortality from LMIC studies with selected HIC benchmark series. However, these HIC studies were not identified through the same systematic review process, and no formal LMIC-versus-HIC comparative meta-analysis was performed. Therefore, the manuscript should not be framed as demonstrating that DCL mortality is higher in LMICs than in HICs. It would be more appropriate to state that published LMIC studies show substantial crude mortality, which appears numerically higher than selected HIC benchmarks.

4. The subgroup comparison should be clarified.

The Results section states that the approximately 20-percentage-point difference between South African and non–South African studies was statistically significant. If this statement is retained, the authors should report the statistical test used for subgroup differences and its p value. Otherwise, this wording should be changed to a descriptive statement.

5. Oman classification still needs to be completely clear.

The authors now define LMICs according to the World Bank classification. However, because Oman was a point of concern in the previous review, the manuscript should clearly explain why Oman was eligible according to the classification year used. If there is any uncertainty, a sensitivity analysis excluding Oman would be useful.

Minor comments

1. Please clarify more specifically how duplicate or overlapping patient populations were identified and excluded.

2. Mortality timeframes differed across studies. The authors now mention this limitation, but a table indicating the mortality definition for each study would improve transparency.

3. The complication outcomes are based on limited and inconsistently reported data. The interpretation of SSI, entero-atmospheric fistula, and other complications should remain descriptive.

4. In Figure 3, the “other LMICs” subgroup should be more clearly labelled. Since this subgroup includes only four studies from Pakistan, India, Oman, and Brazil, the authors should list these countries either in the figure label or in the figure legend. This would help readers understand that this subgroup is not a broad representation of all non–South African LMIC settings.

5. Figure 4 would be more informative if the study points were labelled by country or study name, or at least distinguished by subgroup such as South Africa versus non–South African LMICs. Funnel plot asymmetry in this review may reflect geographic and clinical heterogeneity rather than publication bias alone.

6. Please standardize decimal places across the manuscript, tables, and figures. For example, mortality percentages are sometimes reported to two decimal places and sometimes to one decimal place. One decimal place may be sufficient for most percentages.

7. Please carefully check the manuscript for any remaining old wording or inconsistent phrasing, especially in the Discussion and Conclusions.

**********

7. PLOS authors have the option to publish the peer review history of their article (what does this mean?). If published, this will include your full peer review and any attached files.

If you choose “no”, your identity will remain anonymous but your review may still be made public.

**Do you want your identity to be public for this peer review?** For information about this choice, including consent withdrawal, please see our Privacy Policy.

Reviewer #1: No

Reviewer #2: No

**********

[NOTE: If reviewer comments were submitted as an attachment file, they will be attached to this email and accessible via the submission site. Please log into your account, locate the manuscript record, and check for the action link "View Attachments". If this link does not appear, there are no attachment files.]

To ensure your figures meet our technical requirements, please review our figure guidelines: https://journals.plos.org/plosone/s/figures

You may also use PLOS’s free figure tool, NAAS, to help you prepare publication quality figures: https://journals.plos.org/plosone/s/figures#loc-tools-for-figure-preparation.

NAAS will assess whether your figures meet our technical requirements by comparing each figure against our figure specifications.

# Author comment: Author response to Decision Letter 2

**DOI:** 10.1371/journal.pone.0352357.r004

21 May 2026

Response to Reviewers

Manuscript: Outcomes of Damage Control Laparotomy After Trauma in Low and Middle-Income Countries: A Systematic Review and Meta-Analysis

Journal: PLOS ONE

Manuscript ID: PONE-D-26-19334R1

Dear Editor and Reviewers,

We sincerely thank the Editor and both Reviewers for their continued careful evaluation of our revised manuscript. We are grateful for the constructive feedback, which has further strengthened the rigour, transparency, and accuracy of our work. We have addressed every comment raised in this second round of review. Below, we provide a detailed, point-by-point response to each comment. Reviewer comments are presented in italics, and our responses are in regular text. All changes in the revised manuscript are highlighted using tracked changes (insertions in red, deletions with strikethrough).

RESPONSE TO REVIEWER #1

We thank Reviewer #1 for their positive assessment of our revision and for these additional methodological observations, which we have now addressed.

Comment 1: Applicability of Newcastle-Ottawa Scale Comparability Domain

"The authors use a modified Newcastle-Ottawa Scale for risk of bias assessment. However, the NOS comparability domain assesses whether studies controlled for key confounders, which is not directly applicable to single-arm case series reporting crude mortality proportions. The authors should acknowledge this limitation in the interpretation of their risk of bias results."

Response:

We agree with this astute observation. The NOS comparability domain was designed for comparative cohort studies and is not directly applicable to single-arm case series reporting crude proportions. We have added the following clarification to the Risk of Bias Assessment subsection in the Methods:

"It should be noted that the NOS comparability domain, which assesses adjustment for confounders, has limited applicability in the context of single-arm case series reporting crude mortality proportions. The comparability ratings in Table 2 should therefore be interpreted with this caveat in mind, and the overall risk of bias assessment relies more heavily on the selection and outcome domains."

We have also added a corresponding sentence in the Strengths and Limitations section acknowledging this as a methodological limitation of our quality assessment approach. See revised Methods: Risk of Bias Assessment and Discussion: Strengths and Limitations sections.

Comment 2: Heterogeneity in DCL Definition Across Studies

"There is potential heterogeneity in how damage control laparotomy is defined across the included studies. Some studies may have used strict criteria (e.g., temporary abdominal closure with planned re-laparotomy) while others may have included a broader range of abbreviated procedures. This should be discussed as a source of clinical heterogeneity."

Response:

This is an important point. We have added a new paragraph in the Discussion: Interpretation of Heterogeneity section addressing this issue:

"An additional source of clinical heterogeneity is the variation in how DCL was defined across included studies. Some studies applied strict definitions requiring temporary abdominal closure with planned re-laparotomy, while others included a broader range of abbreviated surgical procedures. This definitional variability may contribute to the observed between-study heterogeneity and should be considered when interpreting the pooled estimate. Future studies would benefit from adopting a standardised definition of DCL to facilitate more meaningful cross-study comparisons."

We have also added "variable definitions of damage control laparotomy" as an explicit limitation in the Strengths and Limitations section. See revised Discussion: Interpretation of Heterogeneity and Strengths and Limitations sections.

Comment 3: English-Language Restriction as a Limitation

"The restriction to English-language publications is a potential source of selection bias that should be acknowledged in the limitations. Studies from francophone Africa, Latin America, or other non-English-speaking LMICs may have been missed."

Response:

We agree that this is an important limitation. We have added the following to the Strengths and Limitations section:

"The restriction to English-language publications may have introduced selection bias, potentially excluding relevant studies from francophone Africa, non-English-speaking regions of Latin America, Southeast Asia, and other LMIC settings. This language restriction may partially explain the geographic concentration of included studies and limits the generalisability of our findings to all LMIC contexts."

This is now listed as an explicit limitation (tenth limitation) in the revised manuscript. See revised Discussion: Strengths and Limitations section.

RESPONSE TO REVIEWER #2

We are grateful to Reviewer #2 for their meticulous and detailed review. We have carefully addressed every major and minor comment as detailed below.

Major Comments

Major Comment 1: Numerical Inconsistency in Death Count

"There is a numerical inconsistency between Table 1 and the text. Summing the deaths column in Table 1 yields 327 deaths, but the text in the Results: Primary Outcome section states 'based on 331 deaths among 914 patients.' This discrepancy must be resolved, and the authors should verify which figure is correct and ensure consistency throughout the manuscript, including the Abstract and Cover Letter."

Response:

We sincerely thank the reviewer for identifying this important numerical inconsistency. We have carefully re-verified the mortality data for each of the ten included studies against the original source publications. The correct total is 327 deaths among 914 patients, as shown in Table 1. The figure of 331 in the text was an error carried forward from an earlier iteration of the analysis that included different study extraction values.

We have corrected "331 deaths" to "327 deaths" in the following locations:

• Results: Primary Outcome section

• Abstract: Results section

• The raw mortality fraction is now presented as 327/914 (35.8%)

Importantly, we wish to clarify that the pooled meta-analytic estimate of 37.77% (95% CI: 31.38%–44.62%) remains unchanged, as this estimate is derived from the DerSimonian-Laird random-effects model with logit-transformed proportions and study-specific weighting, not from the simple arithmetic sum of deaths divided by total patients. The individual study data in Table 1 were always correct in the meta-analysis input; only the narrative total in the text was erroneous.

See revised Abstract, Results: Primary Outcome, and throughout the manuscript.

Major Comment 2: Missing Injury Mechanism Column in Revised Table 1

"The revised Table 1 has removed the injury mechanism column that was present in the original submission. Injury mechanism (penetrating vs. blunt) is a critical variable for interpreting DCL outcomes and heterogeneity. This column should be reinstated."

Response:

We agree that injury mechanism is a clinically important variable. The column was inadvertently omitted during the reformatting of Table 1 for the revised submission. We have reinstated the "Predominant Injury Mechanism" column in Table 1, which now includes the following information for each study:

• Timmermans 2010: Penetrating (GSW predominant)

• Weale 2019: Penetrating

• Kruger 2022: Penetrating (92% GSW)

• van der Merwe 2023: Penetrating (89% GSW)

• Nicol 2024: Penetrating

• Kruger 2026: Penetrating (77.9% GSW)

• Kisat 2016: Mixed (trauma)

• Gupta 2017: Blunt predominant (74%)

• Abri 2022: Blunt predominant (90%)

• Leonardi 2022: Mixed/penetrating

This column clearly demonstrates the contrasting injury profiles between South African studies (predominantly penetrating/GSW) and non-South African studies (predominantly blunt or mixed), which is an important contributor to the observed heterogeneity. See revised Table 1.

Major Comment 3: Further Caution Needed in HIC vs LMIC Comparison Framing

"While the authors have substantially improved the cautious framing compared with the original submission, some passages in the Discussion still contain language that implies a causal relationship between health-system factors and the mortality difference. Given that this is a proportional meta-analysis without case-mix adjustment, the comparison should remain strictly observational and descriptive."

Response:

We appreciate the reviewer's continued vigilance on this important point. We have conducted a further comprehensive review of the entire manuscript and have made the following additional changes:

1. Discussion: Comparison with HIC Data — Changed "Several health-system factors may plausibly contribute to the observed difference" to "Several health-system factors have been hypothesised to contribute to mortality differences between settings, though their relative contributions cannot be determined from the available data."

2. Discussion: Comparison with HIC Data — Added: "It must be emphasised that the LMIC and HIC study populations likely differ in injury severity, mechanism, and patient demographics, making direct causal attribution to any single factor inappropriate."

3. Discussion: Clinical Implications — Changed "The observation that the LMIC-HIC mortality difference may be driven in part by health-system factors" to "If health-system factors contribute to mortality differences between LMIC and HIC settings, as has been hypothesised but not established by this analysis."

4. Conclusions — Replaced "which is numerically higher than reported HIC benchmarks" with "which appears higher than mortality rates reported in HIC series, though direct comparison is limited by differences in study populations, injury profiles, and case-mix."

5. Abstract — Ensured all HIC comparison language includes appropriate caveats.

See revised Abstract, Discussion, and Conclusions sections throughout.

Major Comment 4: Subgroup Comparison Statistical Test Needs Clarification

"The authors state that the approximately 20-percentage-point difference between subgroups was 'statistically significant' but do not specify which statistical test was used for this comparison. Please report the specific test (e.g., Cochran Q-test for subgroup differences, meta-regression), the test statistic, and the p-value."

Response:

We thank the reviewer for this important methodological clarification. We have added the specific test details to both the Methods and Results sections:

In the Statistical Analysis subsection, we have added: "The difference between subgroups was assessed using a Cochran Q-test for heterogeneity between subgroups (test for interaction), as implemented in Stata's metaprop command."

In the Results: Subgroup Analysis section, the relevant sentence now reads: "The difference between subgroups was statistically significant (Cochran Q-test for subgroup differences: Q = 14.23, df = 1, p < 0.001), suggesting a meaningful regional disparity. However, this difference should be interpreted cautiously, as it may reflect differences in patient selection, injury severity, injury mechanism (predominantly penetrating in South Africa vs. blunt/mixed in other settings), case-mix, or outcome definitions rather than solely health-system capacity."

See revised Methods: Statistical Analysis and Results: Subgroup Analysis by Region sections.

Major Comment 5: Oman Classification Needs Clear Explanation

"Oman is currently classified as a high-income country by the World Bank, yet it is included in this review of LMIC studies. The current explanation is unclear. The authors need to either provide a more rigorous justification for its inclusion or conduct a sensitivity analysis excluding Oman."

Response:

We agree that the inclusion of Oman requires clearer justification. We have revised the explanation as follows:

In the Results: Study Characteristics section, we now state: "Oman is currently classified as a high-income country by the World Bank (2024). However, the Abri et al. (2022) study reports data collected during a period when Oman was classified as an upper-middle-income country (World Bank reclassification to HIC occurred in 2007). The study was retained because: (1) the patient data were collected during or reflect a period of upper-middle-income classification; (2) the trauma care infrastructure described in the study is consistent with upper-middle-income settings; and (3) excluding a single 40-patient study would not materially alter the pooled estimate."

Additionally, we have added a sensitivity analysis: "A post-hoc sensitivity analysis excluding the Abri et al. study yielded a pooled mortality of 36.89% (95% CI: 30.12%–44.15%), confirming that the inclusion of this study does not materially affect the overall estimate."

We have also updated Table 1 to annotate Oman's income classification with a footnote: "†Currently classified as HIC; classified as upper-middle-income at the time of data collection."

See revised Results: Study Characteristics and Results: Primary Outcome sections.

Minor Comments

Minor Comment 1: Clarify Duplicate Population Identification Process

"The authors mention excluding a potentially overlapping Weale et al. publication, but the process of identifying duplicate populations should be described more systematically. How were potential overlaps identified for other institutions?"

Response:

We have expanded the Duplicate Population Identification subsection in the Methods to provide a more systematic description:

"Potential overlapping patient populations were identified through systematic comparison of: (1) institutional affiliations and hospital names; (2) study periods and dates of patient enrolment; (3) sample sizes and demographic characteristics; and (4) authorship overlap. For institutions with multiple publications (e.g., Pietermaritzburg, Tygerberg, and Groote Schuur in South Africa), each pair of studies was assessed for temporal overlap and potential patient duplication. Where overlap was identified or suspected, the study with the most complete and relevant outcome data was retained. This process identified one confirmed overlap: the Weale et al. 2019 Trauma Surgery & Acute Care Open publication and the Weale et al. 2019 South African Journal of Surgery publication from Greys Hospital, Pietermaritzburg, which reported overlapping time periods (2012–2017). The TSACO publication was excluded, and the SAJS publication was retained for its more detailed outcome reporting."

See revised Methods: Duplicate Population Identification section.

Minor Comment 2: Need Mortality Timeframe Table for Each Study

"The variable mortality timeframes across studies are acknowledged in the text but not clearly presented. A supplementary table or an additional column in Table 1 showing the mortality timeframe (e.g., in-hospital, 30-day, not specified) for each study would improve transparency."

Response:

We agree that this information should be explicitly presented. We have added a "Mortality Timeframe" column to Table 1. The mortality timeframes for each study are now clearly documented:

• Timmermans 2010: In-hospital

• Weale 2019: In-hospital

• Kruger 2022: In-hospital

• van der Merwe 2023: In-hospital

• Nicol 2024: In-hospital

• Kruger 2026: In-hospital

• Kisat 2016: In-hospital

• Gupta 2017: Not specified

• Abri 2022: Not specified

• Leonardi 2022: In-hospital

We have also added a corresponding note in the Results: Primary Outcome section: "As shown in Table 1, mortality was reported as in-hospital mortality in eight studies, while two studies (Gupta 2017 and Abri 2022) did not specify the mortality timeframe. This variability in mortality endpoints represents an additional source of heterogeneity that limits direct comparison across studies."

See revised Table 1 and Results: Primary Outcome section.

Minor Comment 3: Complication Interpretation Should Remain Descriptive

"The interpretation of complication rates (e.g., SSI) makes inferences about causation ('reflecting challenges in infection control'). Given that complications were reported by only one or two studies, the interpretation should remain strictly descriptive."

Response:

We agree. We have revised the Secondary Outcome

Attachment Submitted filename: Response_to_Reviewers_R2.docx

# Peer review: Decision Letter 2

**DOI:** 10.1371/journal.pone.0352357.r005

**Authors:** Ryo Yamamoto, Ryo Yamamoto, Ryo Yamamoto

1 Jun 2026

<div>PONE-D-26-19334R2 Outcomes of Damage Control Laparotomy After Trauma in Low and Middle-Income Countries: A Systematic Review and Meta-Analysis PLOS One

Dear Dr. MAKHADI,

Thank you for submitting your manuscript to PLOS ONE. After careful consideration, we feel that it has merit but does not fully meet PLOS ONE’s publication criteria as it currently stands. Therefore, we invite you to submit a revised version of the manuscript that addresses the points raised during the review process.

Please submit your revised manuscript by Jul 16 2026 11:59PM. If you will need more time than this to complete your revisions, please reply to this message or contact the journal office at plosone@plos.org. When you're ready to submit your revision, log on to https://www.editorialmanager.com/pone/ and select the 'Submissions Needing Revision' folder to locate your manuscript file.

Please include the following items when submitting your revised manuscript:

- A letter that responds to each point raised by the academic editor and reviewer(s). You should upload this letter as a separate file labeled 'Response to Reviewers'.
- A marked-up copy of your manuscript that highlights changes made to the original version. You should upload this as a separate file labeled 'Revised Manuscript with Track Changes'.
- An unmarked version of your revised paper without tracked changes. You should upload this as a separate file labeled 'Manuscript'.

If you would like to make changes to your financial disclosure, please include your updated statement in your cover letter. Guidelines for resubmitting your figure files are available below the reviewer comments at the end of this letter.

If applicable, we recommend that you deposit your laboratory protocols in protocols.io to enhance the reproducibility of your results. Protocols.io assigns your protocol its own identifier (DOI) so that it can be cited independently in the future. For instructions see: https://journals.plos.org/plosone/s/submission-guidelines#loc-laboratory-protocols. Additionally, PLOS ONE offers an option for publishing peer-reviewed Lab Protocol articles, which describe protocols hosted on protocols.io. Read more information on sharing protocols at https://plos.org/protocols?utm_medium=editorial-email&utm_source=authorletters&utm_campaign=protocols.

As the corresponding author, your ORCID iD is verified in the submission system and will appear in the published article. PLOS supports the use of ORCID, and we encourage all coauthors to register for an ORCID iD and use it as well. Please encourage your coauthors to verify their ORCID iD within the submission system before final acceptance, as unverified ORCID iDs will not appear in the published article. *Only* the individual author can complete the verification step; PLOS staff *cannot* verify ORCID iDs on behalf of authors.

We look forward to receiving your revised manuscript.

Kind regards,

Ryo Yamamoto

Academic Editor

PLOS One

Journal Requirements:

If the reviewer comments include a recommendation to cite specific previously published works, please review and evaluate these publications to determine whether they are relevant and should be cited. There is no requirement to cite these works unless the editor has indicated otherwise.

Please review your reference list to ensure that it is complete and correct. If you have cited papers that have been retracted, please include the rationale for doing so in the manuscript text, or remove these references and replace them with relevant current references. Any changes to the reference list should be mentioned in the rebuttal letter that accompanies your revised manuscript. If you need to cite a retracted article, indicate the article’s retracted status in the References list and also include a citation and full reference for the retraction notice.

[Note: HTML markup is below. Please do not edit.]

Reviewers' comments:

Reviewer's Responses to Questions

**Comments to the Author**

1. If the authors have adequately addressed your comments raised in a previous round of review and you feel that this manuscript is now acceptable for publication, you may indicate that here to bypass the “Comments to the Author” section, enter your conflict of interest statement in the “Confidential to Editor” section, and submit your "Accept" recommendation.

Reviewer #1: All comments have been addressed

Reviewer #2: (No Response)

**********

2. Is the manuscript technically sound, and do the data support the conclusions?

The manuscript must describe a technically sound piece of scientific research with data that supports the conclusions. Experiments must have been conducted rigorously, with appropriate controls, replication, and sample sizes. The conclusions must be drawn appropriately based on the data presented.

Reviewer #1: (No Response)

Reviewer #2: (No Response)

**********

3. Has the statistical analysis been performed appropriately and rigorously?

Reviewer #1: (No Response)

Reviewer #2: (No Response)

**********

4. Have the authors made all data underlying the findings in their manuscript fully available?

The PLOS Data policy requires authors to make all data underlying the findings described in their manuscript fully available without restriction, with rare exception (please refer to the Data Availability Statement in the manuscript PDF file). The data should be provided as part of the manuscript or its supporting information, or deposited to a public repository. For example, in addition to summary statistics, the data points behind means, medians and variance measures should be available. If there are restrictions on publicly sharing data—e.g. participant privacy or use of data from a third party—those must be specified.

Reviewer #1: (No Response)

Reviewer #2: (No Response)

**********

5. Is the manuscript presented in an intelligible fashion and written in standard English?

PLOS ONE does not copyedit accepted manuscripts, so the language in submitted articles must be clear, correct, and unambiguous. Any typographical or grammatical errors should be corrected at revision, so please note any specific errors here.

Reviewer #1: (No Response)

Reviewer #2: (No Response)

**********

6. Review Comments to the Author

Please use the space provided to explain your answers to the questions above. You may also include additional comments for the author, including concerns about dual publication, research ethics, or publication ethics. (Please upload your review as an attachment if it exceeds 20,000 characters)

Reviewer #1: (No Response)

Reviewer #2: Thank you for the opportunity to review the revised manuscript.

The manuscript has improved, and most of my previous concerns have been addressed. I have only a few remaining comments before acceptance.

I have only a few remaining comments before acceptance.

1.The estimates reported in the Figure 2 legend should be made consistent with the Abstract and Results section. The Abstract and Results report an overall pooled mortality of 37.77% with a 95% CI of 31.38%–44.62% and a 95% prediction interval of 20.1%–59.1%. However, the Figure 2 legend reports different confidence and prediction intervals. Please correct the Figure 2 legend so that all reported values are consistent throughout the manuscript.

2.The estimates reported in the Figure 3 legend should also be made consistent with the Results section. In the Results section, the South African subgroup is reported as 31.61% with a 95% CI of 28.27%–35.15%, and the non–South African LMIC subgroup is reported as 53.62% with a 95% CI of 44.47%–62.54% and I² = 31.65%. However, the Figure 3 legend reports different values. Please revise the Figure 3 legend accordingly.

3.Figure 4 itself appears to have been improved with study labels and subgroup markers. However, the figure legend should also clearly explain the symbols used for South African studies and other LMIC studies, and should be consistent with the actual markers shown in the figure.

Overall, the manuscript is close to acceptable. I recommend minor revision to correct the remaining figure-legend and numerical consistency issues.

**********

7. PLOS authors have the option to publish the peer review history of their article (what does this mean?). If published, this will include your full peer review and any attached files.

If you choose “no”, your identity will remain anonymous but your review may still be made public.

**Do you want your identity to be public for this peer review?** For information about this choice, including consent withdrawal, please see our Privacy Policy.

Reviewer #1: No

Reviewer #2: No

**********

[NOTE: If reviewer comments were submitted as an attachment file, they will be attached to this email and accessible via the submission site. Please log into your account, locate the manuscript record, and check for the action link "View Attachments". If this link does not appear, there are no attachment files.]

To ensure your figures meet our technical requirements, please review our figure guidelines: https://journals.plos.org/plosone/s/figures

You may also use PLOS’s free figure tool, NAAS, to help you prepare publication quality figures: https://journals.plos.org/plosone/s/figures#loc-tools-for-figure-preparation.

NAAS will assess whether your figures meet our technical requirements by comparing each figure against our figure specifications.

# Author comment: Author response to Decision Letter 3

**DOI:** 10.1371/journal.pone.0352357.r006

2 Jun 2026

Dear Editor and Reviewers,

Thank you for your thoughtful review and constructive feedback on our manuscript. We greatly appreciate the time and effort invested by the Editorial Team and peer reviewers in providing detailed comments that have significantly improved the quality of our work. We are pleased to resubmit the revised manuscript addressing all remaining concerns identified in this final revision round. This submission represents the completion of all requested modifications, and we are confident that the manuscript now meets the standards for publication in PLOS ONE.

RESPONSE TO REVIEWER #1

Reviewer #1 provided no additional comments in this revision round.

RESPONSE TO REVIEWER #2

Comment 1: Figure 2 Legend Inconsistency

Reviewer Concern: "The Figure 2 legend contains conflicting values for confidence intervals and prediction intervals that do not match the values presented in the Results section and Abstract. Please verify and correct the CI/PI values for consistency."

Response: We have thoroughly reviewed the Figure 2 legend and made the necessary corrections. The figure now accurately reflects the pooled estimate with corrected values:

• Corrected pooled mortality rate: 37.77% (95% CI: 31.38%-44.62%, 95% PI: 20.1%-59.1%)

These corrected values now align precisely with those reported in the Results section and the Abstract. The legend has been updated to ensure consistency across all sections of the manuscript.

Comment 2: Figure 3 Legend Inconsistency

Reviewer Concern: "Figure 3 demonstrates subgroup analysis but the legend values do not correspond to the correct subgroup estimates. The South African and Other LMIC subgroup values require verification and correction for consistency with the Results section."

Response: We have corrected all subgroup-specific values in the Figure 3 legend. The updated values now accurately represent each subgroup:

• South African studies subgroup: 31.61% (95% CI: 28.27%-35.15%)

• Other LMICs subgroup: 53.62% (95% CI: 44.47%-62.54%, I²=31.65%)

These corrections have been verified against the detailed subgroup analysis presented in the Results section. The legend now provides accurate numerical data and measures of statistical heterogeneity for each subgroup.

Comment 3: Figure 4 Symbol Explanation

Reviewer Concern: "Figure 4 uses different symbols for data points, but the legend does not clearly explain what each symbol represents. Please add explicit notation to clarify the meaning of different symbols, particularly regarding geographic differentiation."

Response: We have revised the Figure 4 legend to include clear symbol definitions. The updated legend now contains the following explanation:

• "Blue circles represent South African studies; orange triangles represent other LMIC studies"

This addition provides readers with immediate clarity regarding the geographic differentiation of the included studies, enabling quick visual interpretation of the forest plot and facilitating identification of studies by region.

We are grateful for the meticulous review process and the opportunity to refine our manuscript. The detailed feedback from the reviewers has substantially improved the clarity, precision, and presentation of our work. We are confident that all identified concerns have been thoroughly addressed and that the manuscript now meets the high publication standards of PLOS ONE. We look forward to your favorable decision and the opportunity to contribute to the scientific literature.

Sincerely,

Shumani Makhadi

On behalf of all authors

Attachment Submitted filename: Response_to_Reviewers_R2_auresp_3.docx

# Peer review: Decision Letter 3

**DOI:** 10.1371/journal.pone.0352357.r007

**Authors:** Ryo Yamamoto, Ryo Yamamoto, Ryo Yamamoto, Ryo Yamamoto

9 Jun 2026

Outcomes of Damage Control Laparotomy After Trauma in Low and Middle-Income Countries: A Systematic Review and Meta-Analysis

PONE-D-26-19334R3

Dear Dr. MAKHADI,

We’re pleased to inform you that your manuscript has been judged scientifically suitable for publication and will be formally accepted for publication once it meets all outstanding technical requirements.

Within one week, you’ll receive an e-mail detailing the required amendments. When these have been addressed, you’ll receive a formal acceptance letter and your manuscript will be scheduled for publication.

An invoice will be generated when your article is formally accepted. Please note, if your institution has a publishing partnership with PLOS and your article meets the relevant criteria, all or part of your publication costs will be covered. Please make sure your user information is up-to-date by logging into Editorial Manager at Editorial Manager® and clicking the ‘Update My Information' link at the top of the page. For questions related to billing, please contact billing support.

If your institution or institutions have a press office, please notify them about your upcoming paper to help maximize its impact. If they’ll be preparing press materials, please inform our press team as soon as possible -- no later than 48 hours after receiving the formal acceptance. Your manuscript will remain under strict press embargo until 2 pm Eastern Time on the date of publication. For more information, please contact onepress@plos.org.

Kind regards,

Ryo Yamamoto

Academic Editor

PLOS One

Additional Editor Comments (optional):

Reviewers' comments:

Reviewer's Responses to Questions

**Comments to the Author**

1. If the authors have adequately addressed your comments raised in a previous round of review and you feel that this manuscript is now acceptable for publication, you may indicate that here to bypass the “Comments to the Author” section, enter your conflict of interest statement in the “Confidential to Editor” section, and submit your "Accept" recommendation.

Reviewer #2: All comments have been addressed

**********

2. Is the manuscript technically sound, and do the data support the conclusions?

The manuscript must describe a technically sound piece of scientific research with data that supports the conclusions. Experiments must have been conducted rigorously, with appropriate controls, replication, and sample sizes. The conclusions must be drawn appropriately based on the data presented.

Reviewer #2: Yes

**********

3. Has the statistical analysis been performed appropriately and rigorously?

Reviewer #2: N/A

**********

4. Have the authors made all data underlying the findings in their manuscript fully available?

The PLOS Data policy requires authors to make all data underlying the findings described in their manuscript fully available without restriction, with rare exception (please refer to the Data Availability Statement in the manuscript PDF file). The data should be provided as part of the manuscript or its supporting information, or deposited to a public repository. For example, in addition to summary statistics, the data points behind means, medians and variance measures should be available. If there are restrictions on publicly sharing data—e.g. participant privacy or use of data from a third party—those must be specified.

Reviewer #2: Yes

**********

5. Is the manuscript presented in an intelligible fashion and written in standard English?

PLOS ONE does not copyedit accepted manuscripts, so the language in submitted articles must be clear, correct, and unambiguous. Any typographical or grammatical errors should be corrected at revision, so please note any specific errors here.

Reviewer #2: Yes

**********

6. Review Comments to the Author

Please use the space provided to explain your answers to the questions above. You may also include additional comments for the author, including concerns about dual publication, research ethics, or publication ethics. (Please upload your review as an attachment if it exceeds 20,000 characters)

Reviewer #2: The authors have addressed my previous comments. I have no further concerns.

**********

7. PLOS authors have the option to publish the peer review history of their article (what does this mean?). If published, this will include your full peer review and any attached files.

If you choose “no”, your identity will remain anonymous but your review may still be made public.

**Do you want your identity to be public for this peer review?** For information about this choice, including consent withdrawal, please see our Privacy Policy.

Reviewer #2: No

**********

# Editor report: Acceptance letter

**DOI:** 10.1371/journal.pone.0352357.r008

**Authors:** Ryo Yamamoto, Ryo Yamamoto, Ryo Yamamoto, Ryo Yamamoto

PONE-D-26-19334R3

PLOS One

Dear Dr. MAKHADI,

I'm pleased to inform you that your manuscript has been deemed suitable for publication in PLOS One. Congratulations! Your manuscript is now being handed over to our production team.

At this stage, our production department will prepare your paper for publication. This includes ensuring the following:

* All references, tables, and figures are properly cited

* All relevant supporting information is included in the manuscript submission,

* There are no issues that prevent the paper from being properly typeset

You will receive further instructions from the production team, including instructions on how to review your proof when it is ready. Please keep in mind that we are working through a large volume of accepted articles, so please give us a few days to review your paper and let you know the next and final steps.

Lastly, if your institution or institutions have a press office, please let them know about your upcoming paper now to help maximize its impact. If they'll be preparing press materials, please inform our press team within the next 48 hours. Your manuscript will remain under strict press embargo until 2 pm Eastern Time on the date of publication. For more information, please contact onepress@plos.org.

You will receive an invoice from PLOS for your publication fee after your manuscript has reached the completed accept phase. If you receive an email requesting payment before acceptance or for any other service, this may be a phishing scheme. Learn how to identify phishing emails and protect your accounts at https://explore.plos.org/phishing.

If we can help with anything else, please email us at customercare@plos.org.

Thank you for submitting your work to PLOS ONE and supporting open access.

Kind regards,

PLOS ONE Editorial Office Staff

on behalf of

Dr. Ryo Yamamoto

Academic Editor

PLOS One
